# *Mycobacterium tuberculosis* small RNAs MTS0997 and MTS1338 regulate both pathogen virulence and host immune responses

**DOI:** 10.3389/fcimb.2026.1918881

**Published:** 2026-09-28

**Authors:** Elena G. Salina, Artem Grigorov, Billy Martini, Yulia Skvortsova, Nadezhda Logunova, Marina Kapina, Mikhail Shumkov, Oksana Bychenko, Elena Svirshchevskaya, Irina Bocharova, Konstantin Majorov, Irina Linge, Alexander Apt, Tatyana Azhikina

**Affiliations:** 1 Shemyakin-Ovchinnikov Institute of Bioorganic Chemistry of the Russian Academy of Sciences, Moscow, Russia; 2 Bach Institute of Biochemistry, Research Center of Biotechnology of the Russian Academy of Sciences, Moscow, Russia; 3 Immunology Department, Central TB Research Institute, Moscow, Russia

**Keywords:** gene expression profiling, infection *in vitro*, infection *in vivo*, mouse model, *Mycobacterium tuberculosis*, small RNAs, virulence

## Abstract

Previously, others and we demonstrated that small RNAs MTS1338 and MTS0997 of *Mycobacterium tuberculosis*, the causative agent of tuberculosis (TB), are essential for persistence inside host macrophages. However, their role in mycobacterial transcriptional profile regulation was characterized incompletely, and potential influence on host immune responses and the outcome of infection remained obscure. To obtain new insights in *M. tuberculosis* biology and TB pathogenesis we established unmarked single-knockout (KO) *M. tuberculosis* strains with deleted genes for MTS0997, or MTS1338, as well as the double KO strain. These strains, their parental wild type progenitor and complemented double KO variant were explored in three experimental settings: (i) whole genome expression profiling during cultural growth, (ii) whole genome transcriptional analysis of murine bone marrow-derived macrophages activated or not with IFN-γ, and (iii) TB infection in genetically highly TB-susceptible mice. The results of our experiments indicate that deletion of MTS0997 sRNA results in a more pronounced impact on *M. tuberculosis* biology compared to MTS1338. The most prominent phenotypic shifts in *MTS0997*-deleted strain were modulation of bacterial succinate/fumarate respiration, up-regulation of genes belonging to the ESX-1 secretion system, increase in pathogen virulence for the murine host and decreased production of a few pro-inflammatory cytokines in the lung tissue. In addition, differences in the sizes of B-cell and other MHC-II-expressing cell populations in lungs of mice infected with single- compared to double-KO strains provided evidence for transcriptional epistasis, a phenomenon that remains poorly understood in mycobacteria.

## 1 Introduction

Among other functions, small bacterial RNAs (sRNAs) procure rapid molecular switches allowing for synchronization of pathogens’ physiological states with hostile environment within the host. Their base-pairing with target mRNAs serves for fine-tuning the expression levels of genes determining virulence, stress responses, immune evasion and adaptation (Djapgne and Oglesby, 2021; Skvortsova et al., 2025). For *Mycobacterium tuberculosis*, the causative agent of tuberculosis (TB), some sRNAs, such as MTS1338 (DrrS) and MTS0997 (Mcr11), are essential for persistence inside macrophages (Garg et al., 2024; Ignatov et al., 2014; Kumar and Dutta, 2024; Ostrik et al., 2021). For instance, MTS1338, a component of the DosR dormancy regulon, is involved not only in the arrest of phagosome maturation but also in modulation of host cytokine profiling, thereby creating a protective niche for the pathogen (Bychenko et al., 2021; Salina et al., 2019; Schwenk and Arnvig, 2018). Similarly, an increase in the MTS0997 expression under acidic stress conditions shifts metabolism towards fatty acid utilization critical for long-term survival within granuloma (Alvarez-Eraso et al., 2022).

Earlier, others and we demonstrated that MTS1338 and MTS0997 are highly expressed during the stationary phase of culture growth and under stressful conditions, e.g., hypoxia, potassium deficiency and macrophage-related stresses, *in vitro* and *in vivo* (Arnvig and Young, 2012; Ignatov et al., 2015; Martini et al., 2023; Salina et al., 2019; Singh and Dutta, 2024). Presently available data suggest that both sRNAs may play important roles in TB pathogenesis. In the present study, we established unmarked single-knockout (KO) *M. tuberculosis* strains with deleted *MTS0997* (d_0997 strain) or *MTS1338* (d_1338 strain) genes, as well as the double KO strain (dd_0997_1338). We characterized the effects of MTS0997 and MTS1338 deletion on mycobacterial and host gene expression. Comparing the single and double mutants enabled us to assess the possibility of functional redundancy of these two sRNAs, and their potential genetic interactions. Together, the results obtained provide strong evidence that specific *M. tuberculosis* sRNAs regulate both mycobacterial virulence and certain components of host inflammatory and immune responses.

## 2 Materials and methods

### 2.1 Bacterial strains, media, and growth conditions

*M. tuberculosis* H37Rv strain was obtained from the bacterial collection of the Bach Institute of Biochemistry (Research Center of Biotechnology of the Russian Academy of Sciences, Moscow, Russia) and *M. tuberculosis* mutant strains were obtained in this study. Bacteria from frozen stocks were pre-cultured in Middlebrook 7H9 medium (Himedia, India) with 10% ADC growth supplement (Himedia) and 0.05% Tween 80 (NeoFroxx GmbH, Germany) at 37 °C with agitation (200 rpm) for 10–12 days and then cultured in the same medium for another 7–9 days. In all experiments, the numbers of viable mycobacteria (CFUs) were determined by plating 10-fold serial dilutions of liquid cultures or organ homogenates in triplicate onto 7H10 agar supplemented with ADC (*in vitro* experimentation performed at the Bach Institute of Biochemistry) or Dubos agar supplemented with ADC (experiments *in vivo* performed at the Central TB Institute) and incubated at 37 °C for 21 days.

### 2.2 Construction of KO and complemented *M. tuberculosis* strains

The allelic replacement technique was used to generate *M. tuberculosis* KO d_0997 and d_1338 strains as described previously (Parish and Stoker, 2000). Briefly, homologous regions flanking the *MTS0997* gene (1280 bp left, 2716 bp right) were amplified from genomic DNA using primers 0997_FR1_For/0997_FR1_Rev and 0997_FR2_For/0997_FR2_Rev. The PCR products were inserted into the p2NIL delivery plasmid at *Bam*HI/*Xba*I and *Xba*I/*Not*I restriction sites, respectively. Homologous regions flanking the *MTS1338* gene (3076 bp left, 1112 bp right) were also amplified from genomic DNA using primers 1338_FR1_For/1338_FR1_Rev and 1338_FR2_For/1338_FR2_Rev, and the PCR products were inserted into the p2NIL delivery plasmid at *Sca*I/*Bam*HI and *Bam*HI/*Not*I restriction sites, respectively. The *sacB*, *hygR*, and *lacZ* selection genes from the pGOAL19 vector were inserted into p2NIL at the *Pac*I restriction site to yield the p2NIL_d_0997 and p2NIL_d_1338 suicide plasmids (**Supplementary Figure 1**), which were used to transform electro-competent *M. tuberculosis* H37Rv cells using a BioRad Gene Pulser (USA) in a cuvette with 2 mm electrode spacing; pulse parameters: resistance 1000 Ω, voltage 2.5 kV, capacitance 25 µF (exponential protocol).

During the first homologous recombination, clones were selected on a medium containing 5-bromo-4-chloro-3-indolyl-β-D-galactopyranoside (X-gal, 50 μg/mL) and kanamycin (50 μg/mL) or hygromycin (50 μg/mL). To obtain double crossovers, the resulting colonies were incubated in Middlebrook 7H9 medium with 10% ADC and 0.05% Tween 80 at 37 °C without antibiotics and plated onto 7H10/ADC agar containing sucrose (2% w/v) and X-gal (50 μg/mL). White colonies were screened for the absence of resistance to hygromycin and kanamycin, and selected colonies were checked for gene deletion by PCR with primers test_d0997_for/test_d0997_rev and test_d1338_for/test_d1338_rev, respectively (**Supplementary Figure 2**).

The double-KO mutant dd_0997_1338 with the two deleted genes (*MTS1338* and *MTS0997*) was produced similarly using the d_0997 mutant as a parental strain (**Supplementary Figure 3**). MTS1338 deletion was achieved using the p2NIL_d_1338 suicide plasmid.

The absence of *MTS1338* and *MTS0997* in d_0997, d_1338, and dd_0997_1338 strains was confirmed by whole genome sequencing; the mutant strain data can be accessed from NCBI SRA (Bioproject ID: PRJNA701202).

For the complementation of the dd_0997_1338 strain, electro-competent cells were transformed with the pMV306_0997_1338 integrating plasmid containing both the *MTS1338* and *MTS0997* genes under the *rrnB* mycobacterial promoter. Control wt_empty and dd_0997_1338_empty strains were produced by transformation of the wt and dd_0997_1338 strains with pMV306_rrnB empty vector.

Oligonucleotides, plasmids, and strains used are listed in **Supplementary Table 1**.

### 2.3 *M. tuberculosis* passage *in vivo*

To restore full virulence in all *M. tuberculosis* strains, mycobacteria were incubated under standard culture conditions to reach mid-logarithmic phase (optical density (OD)_600_ = 0.6 – 0.8), washed with PBS, re-suspended in 0.9% NaCl solution, and injected intravenously into C57BL/6JYCit (B6) mice (10^5^ bacteria per animal). At day 21 post infection, mice were sacrificed, and 50 μL of serial 10-fold dilutions of spleen homogenates were plated on Dubos agar with 10% of ADC growth supplement and incubated for 21 days at 37 °C. The obtained colonies were re-suspended and cultured in Middlebrook 7H9 medium supplemented with ADC and Tween 80 until OD_600_ = 2–3; then, culture aliquots were frozen in medium containing 50% glycerol and stored at -70 °C.

### 2.4 *M. tuberculosis* RNA isolation

Bacterial cultures were grown in Middlebrook 7H9 medium supplemented with ADC and Tween 80 to OD_600_ ~1.0 and centrifuged at 4 °C, 4000 rpm for 15 min. Pellets were washed twice with fresh medium, rapidly cooled on ice, and centrifuged again. Total RNA was isolated as described earlier (Rustad et al., 2009). To achieve mechanical lysis of the robust *M. tuberculosis* cell wall, cells suspended in Trizol reagent (Life Technologies, USA) were subjected to high-speed reciprocal agitation for 30 s with 0.1-mm zirconium beads at a shaking speed of 4,350 rpm using a BeadBeater (BioSpec Products, Bartlesville, OK, USA). The procedure was repeated three times, with the samples cooled on ice between cycles. After phenol-chloroform extraction, the RNA samples were treated with Turbo DNase (Life Technologies, Carlsbad, CA, USA) to remove traces of genomic DNA. RNA quantity and purity were determined on a spectrophotometer, and its integrity was assessed by electrophoresis in 1% agarose gel and confirmed using Tape Station 2200 (Agilent Technologies, Santa Clara, CA, USA).

### 2.5 Macrophage culture and infection

BMDMs were generated in the presence of L929-conditioned medium as a source of M-CSF (LCCM) as described earlier (Marim et al., 2010). Briefly, bone marrow was flushed from femurs and tibias of C57BL/6 mice, single cell suspensions were obtained by passage through a 25-gauge needle, and erythrocytes were lysed with ACK buffer (150 mM NH_4_CL, 10 mM KHCO_3_, 0.1 mM EDTA). Cells were cultured in six-well tissue culture plates (3–5×10^6^ cells/well) in RPMI medium containing 10% FCS, 30% LCCM, 100 U/ml penicillin, 100 µg/ml streptomycin, and 2 mM L-glutamine (PanEco, Russia), with medium replacement every 2 days. At day 6, adhered cells were used for further experiments.

*M. tuberculosis* strains were grown until early logarithmic phase (OD_600_ = 0.8–1.0), centrifuged at 4000 g, washed with PBS, and re-suspended in RPMI-1640 medium containing 5% FCS. To break up bacterial aggregates, *M. tuberculosis* culture was homogenized by serially passing it through a 25-gauge needle. BMDMs cultured in 25 cm^2^ flasks were activated or not with 100 U/ml IFN-γ (Sigma, St. Louis, MO, USA) for 24 h and infected with mycobacterial strains (MOI 2:1) for 4 h at 37 °C. Cultures were washed three times with PBS to remove free mycobacteria and further incubated in fresh RPMI-1640 medium with 5% FCS at 37 °C in a CO_2_-incubator for 24 h. BMDMs were washed with PBS and re-suspended in 1 ml of Trizol (Invitrogen, USA) for RNA isolation.

### 2.6 cDNA synthesis and qRT-PCR

cDNA was synthesized from 1 mg of total RNA using either random hexanucleotides for bacterial RNA or oligo(dT) primer for murine RNA and SuperScript III reverse transcriptase (Life Technologies) according to the manufacturer’s protocol. For each sample, identical “Minus-RT” control reactions were prepared by omitting the reverse transcriptase enzyme to serve as negative controls for genomic DNA contamination. The cDNA samples served as templates for qRT-PCR performed with qPCRmix-HS SYBR (Evrogen) in a LightCycler 480 Real-Time PCR system (Roche, Switzerland) at the following conditions: 40 cycles at 95 °C for 20 s, 60 °C for 20 s, and 72 °C for 30 s. Three biological and nine technical replicates were used to ensure reproducibility, and the results were analyzed with LinRegPCR v 2014.6. The data were normalized against 16S rRNA (bacteria) or β-actin (mice) to correct for sample-to-sample variations, and relative expression ratios were calculated as described earlier (Ganger et al., 2017).

### 2.7 Libraries for RNA-Seq and data analyses

RNA samples from mycobacterial strains were depleted of rRNA using the NEBNext rRNA Depletion Kit (Bacteria). Poly(A) fractions of BMDM RNA were isolated with NEBNext Poly(A) mRNA Magnetic Isolation Module (New England BioLabs, USA). Depleted RNA samples were used to generate sequencing libraries with the NEBNext Ultra II Directional RNA Library Prep Kit (New England BioLabs) according to the manufacturer’s protocol. Sequencing was performed in triplicate using the Illumina NovaSeq6000 (SP-type, 150 bp paired-end reads; Illumina, San Diego, CA, USA).

Bacterial transcriptomics: After quality control evaluation, the reads were mapped on the reference *M. tuberculosis* H37Rv genome (GenBank accession number AL123456.3) using Bowtie2 (Langmead et al., 2019). Gene-level read counts were generated with featureCounts (Liao et al., 2014), which assigns mapped reads to annotated genomic features. Differential expression analysis was performed using the DESeq2 software package (Love et al., 2014). Genes were considered differentially expressed at adjusted p-value < 0.01 and |log_2_FC| > 1.0. Functional pathway enrichment analysis of differentially expressed *M. tuberculosis* genes was performed using KEGG pathway annotations for *M. tuberculosis* H37Rv. Pathway overrepresentation was tested using Fisher’s exact test against the background of KEGG-annotated genes, followed by Benjamini-Hochberg correction for multiple testing.

Mouse transcriptomics: After quality control evaluation using FastQC, RNA-seq reads were quantified with Salmon (Patro et al., 2017) against the *Mus musculus* reference transcriptome from GENCODE Mouse Release M31. Transcript-level abundance estimates from Salmon quantification files were imported into R using the tximport package and analyzed for differential expression using DESeq2 (Love et al., 2014). Standard DESeq2 normalization was applied, and differential expression was assessed using the negative binomial model. Differentially expressed genes were identified using an adjusted *P*-value cutoff of 0.01 after Benjamini-Hochberg correction and an absolute log_2_ fold-change threshold of 1. Results without log_2_FC shrinkage were analyzed independently.

GO Biological Process enrichment analysis was performed in R using clusterProfiler and the org.Mm.eg.db annotation database. Upregulated and downregulated genes in IFN-gamma activated BMDMs infected with dd_0997_1338 compared with WT were analyzed separately. Differentially expressed genes were defined as genes with adjusted p-value ≤ 0.01 and |log_2_FoldChange| > 1. Entrez IDs were used for enrichment analysis, with all mapped genes from the differential expression table used as the background universe. P-values were adjusted using the Benjamini-Hochberg method, and redundant GO terms were collapsed using a semantic similarity cutoff of 0.8. For visualization, the top 10 enriched GO Biological Process terms were selected for each gene group based on adjusted p-value.

All RNAseq data generated in this study are publicly available in the Gene Expression Omnibus (GEO) database under accession numbers GSE330228 and GSE220660.

### 2.8 Mouse TB model

Mice of the I/StSnEgYCit (I/St) strain were housed under conventional, non-SPF conditions in the Animal Facilities of the Central Research Institute of Tuberculosis (CIT, Moscow, Russia) in accordance with the guidelines from the Russian Ministry of Health # 755, and under the NIH Office of Laboratory Animal Welfare (OLAW) Assurance #A5502-11. Female mice aged 2.5–3.0 months were used. Experimental procedures for this study were approved by the CTRI IACUC (protocol 3 of March 6, 2024).

Mycobacterial samples for mouse infection were prepared exactly as described previously (Eruslanov et al., 2004). Briefly, *M. tuberculosis* strains were incubated under standard culture conditions to optical density (OD)_600_ = 0.8–1.0, washed with PBS, re-suspended in 0.9% NaCl solution, and mice were infected *via* respiratory tract with ∼100 viable CFU/mouse using an Inhalation Exposure System (Glas-Col, USA), as described (Radaeva et al., 2005, 2008). The size of challenging dose was confirmed in preliminary experiments by plating serial 2-fold dilutions of 2-ml homogenates of the whole lungs obtained from I/St females at 2 h post-exposure onto Dubos agar and counting colonies after 3-wk incubation at 37 °C. Survival time was monitored daily starting on day 50 post-infection.

### 2.9 Lung cell preparations and flow cytometry

Lungs from individual mice were enzymatically digested exactly as described previously (Eruslanov et al., 2004). Briefly, blood vessels were washed out by perfusion with 0.02% EDTA-PBS through the right ventricle and cut *vena cava.* To extract total RNA, R2 and R4 lung lobes of each infected mouse were homogenized in Trizol reagent (Life Technologies, USA), and RNA was isolated according to a standard phenol-chloroform protocol followed by DNAse treatment (Turbo DNase, Life Technologies). R1 and R3 lung lobes were removed, sliced into 1–2 mm^3^ pieces and incubated at 37 °C for 90 min in supplemented RPMI-1640 containing 200 U/mL collagenase and 50 U/mL DNase-I (Sigma, USA). Single-cell suspensions from 9 mice were obtained individually. To assess CFU counts 200 µl from each suspension was collected and 10-fold serial dilutions were plated on Dubos agar and incubated at 37 °C for 20–22 days. The remaining single cell suspensions were washed twice in HBSS containing 2% FCS and antibiotics. Cell phenotypes were analyzed by flow cytometry using FACS Canto II machine (BD Biosciences, San Jose, CA, USA). The following labeled monoclonal antibodies were used for staining: CD4-Pe-Cy7 (GK1.5), CD8-PerCp (53–6.7), CD19-BV510 (6D5), Ly6G-APC-Cy7 (1A8, BD) CD11b-BV421 (M1/70), and CD11c-APC (N418) (all from BioLegend, Heidelberg, Germany), and I-Ap-FITC (BD Biosciences).

### 2.10 Statistical analysis

Statistical analysis was performed with ANOVA test and Student’s t-test using GraphPad Prism8.0 software (GraphPad Software, San Diego, CA). All results were presented as the mean ± SD of three independent experiments. P < 0.05 was considered to indicate statistically significant differences.

## 3 Results

### 3.1 Establishment of *M. tuberculosis* strains d_0997, d_1338 and dd_0997_1338 lacking genes for corresponding sRNAs

To develop unmarked KO strains, we explored the pNIL/pGOAL allele replacement procedure widely used in mycobacterial genetic engineering [(Parish and Stoker, 2000), **Supplementary Figure 1**]. Homologous regions flanking the *MTS1338* gene (1.957.600-1.960.675bp – left, 1.960.768-1.961.879bp – right) and *MTS0997* gene (1.411.823-1.413.102bp – left and 1.413.216-1.415.931bp – right) (see *Mycobacterium tuberculosis* H37Rv complete genome; Ref seq 000962.3, Sequence ID: AL123456.9), were amplified and cloned into the p2NIL vector. To produce double crossovers, we applied the two-step strategy basing on kanamycin, hygromycin, and X-gal selection at the plasmid integration stage with subsequent sucrose/X-gal selection, and kanamycin/hygromycin counter-selection. Resulting clones were checked for the absence of *MTS1338* or *MTS0997* by PCR (**Supplementary Figure 2**) with confirmation by Sanger sequencing of corresponding PCR products, and by the whole genome sequencing. Double KO mutant lacking both *MTS1338* and *MTS0997* was produced similarly by deleting *MTS1338* gene in the d_0997 strain (**Supplementary Figure 3**).

To restore a high level of virulence in the strains that underwent genetic manipulations and/or multiple cultural passages, KO and wild type strains were passaged through C57BL/6 mice using the following protocol (Grosset and Ji, 1998): intravenous infection, 21d resting period, seeding of spleen homogenates on Middlebrook agar, preparation of log-phase mycobacterial aliquots for the storage at -80 °C. To check whether or not recombination events altered the growth rate of KO strains, we compared the speed of growth of novel and the wild type H37Rv strains in 7H9 medium supplemented with ADC and Tween-80 and detected no inter-strain differences (**Supplementary Figure 4**).

### 3.2 Transcriptional changes in KO mycobacterial strains

#### 3.2.1 General features

To determine whether sRNA deletions resulted in altered gene expression profiles, we performed transcriptome sequencing of the wild type and three KO strains. Sequencing reads were mapped against the reference *M. tuberculosis* H37Rv complete genome, the details of mapping are provided in **Supplementary Table 2**. Genes were considered differentially expressed at the |log_2_FC| ≥ 1, p.adj ≤ 0.01. The list of differentially expressed genes (DEGs) is provided in **Supplementary Table 2**.

Principal component analysis (PCA) confirmed acceptable technical quality of the obtained transcriptomes and revealed unique transcriptional clusters in KO compared to the wild type strain (**Figure 1A**). Transcripts of the wild type and d_1338 strains largely clustered together; however, those of d_0997 and dd_0997_1338 strains clearly differed: only two DEGs were found in d_1338 KO transcriptome, whereas 33 DEGs (20 up-regulated, 13 down-regulated) were found in d_0997 and 52 (28 up-regulated, 24 down-regulated) in dd_0997_1338 strain. As shown at the Venn diagram (**Figure 1B**), gene expression profiles of d_0997 and dd_0997_1338 essentially overlapped, providing 30 common DEGs. **Figure 1C** provides the heat-map of expression levels for selected genes involved in respiratory and energy metabolism, lipid metabolism, amino acid biosynthesis and virulence factors secretion, some of which were differentially expressed across individual biological replicates (p.adj ≤ 0.01; |log_2_FC| ≥ 1). Although moderate gene-specific variation was observed among individual biological replicates, the overall expression pattern of the selected DEGs remained strain-associated, with broadly concordant changes in the d_0997 and dd_0997_1338 strains. Functional analysis of the affected biological pathways using DAVID Bioinformatics Resources revealed the most significantly up-regulated (**Figure 1D**) and down-regulated (**Figure 1E**) pathways. Interestingly, the expression of only two genes was up-regulated in all three KO *M. tuberculosis* strains, both belonging to the regulon encoding components of succinate dehydrogenase – Rv0247c (>10-fold) and Rv0248c (7-fold). Succinate oxidation is the major contributor of electrons to the respiratory chain and is required for optimal growth of *M. tuberculosis* both on fermentable and non-fermentable carbon sources (Adolph et al., 2022).

**Figure 1:**
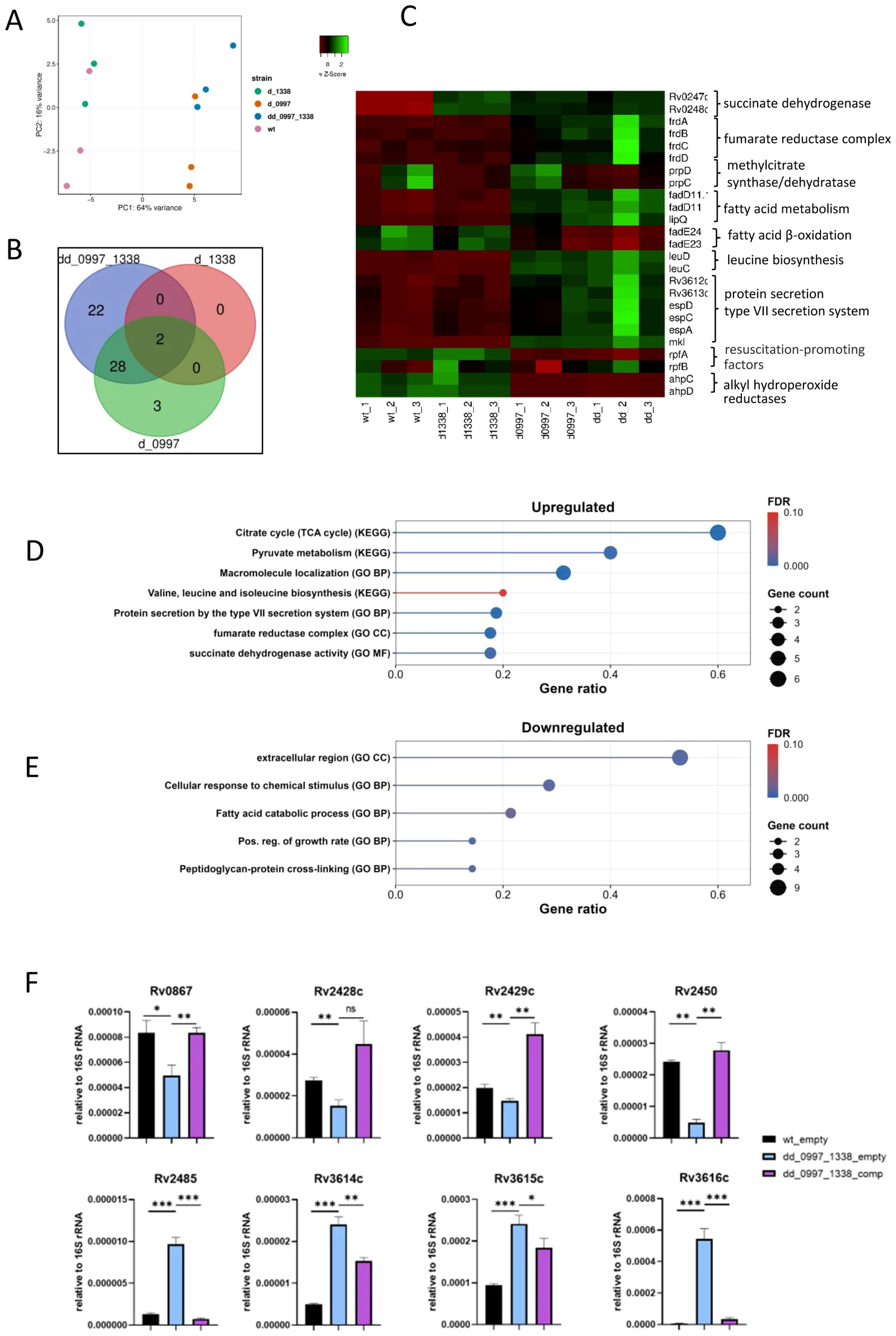
Transcriptional changes caused by sRNA KO in *M. tuberculosis*. **(A)** PCA plot showing the difference between KO strains and wild type mycobacteria. **(B)** Venn diagram of DEGs in KO strains compared with the wild type strain. **(C)** Heatmap showing DEGs related to respiratory and energy metabolism, lipid metabolism, amino acid biosynthesis, and virulence factor secretion in individual biological replicates (p < 0.05; |log_2_FC| ≥ 1). Functional groups of genes are given. **(D, E)** Functional category enrichment analysis of genes upregulated **(D)** and downregulated **(E)** in the double-KO strain compared to the wild type strain. Lollipop plots show significantly enriched metabolic pathways obtained using DAVID bioinformatics resources. The size of each bubble indicates the number of genes associated with the respective pathway, whereas color intensity represents statistical significance based on the false discovery rate (FDR). **(F)** Validation of DEGs by qRT-PCR. mRNA expression was determined in the wt_empty, dd_0997_1338_ empty, dd_0997_1338_ comp (complementation of both sRNA genes) strains in the mid-log phase and normalized to that of 16S rRNA. *p < 0.05, **p < 0.01, and ***p < 0.001. The data are presented as the mean ± SD of three biological replicates for each strain.

#### 3.2.2 Deletion of the sRNA *MTS0997* gene: consequences

The increased expression of the *frdABCD* locus in d_0997 and dd_0997_1338 strains is potentially an important phenotype. This locus encodes fumarate reductases, the enzymes allowing for usage fumarate as an electron acceptor instead of oxygen thus supporting anaerobic respiration and growth under conditions of limited oxygen access inside host macrophages. By activating the Frd pathway *M. tuberculosis* maintains membrane potential and produce enough ATP to survive in dormant state (Schwarz et al., 2021).

We observed significant increase in the expression levels of *espA*, *espC*, and *espD* genes encoding secreted proteins of the ESX-1 system in dd_0997_1338 and d_0997 compared to the wild type strain. EspA is essential for secretion of major *M. tuberculosis* virulence factors EsxA (ESAT-6) and EsxB (cfp10) and is involved in mycobacterial release from phagolysosomes into cytoplasm (Chen et al., 2010; Garces et al., 2010). EspC is required for regulation of EspA expression, whilst EspD stabilizes EspA/EspC complex and maintains optimal levels of EspA and EspC within the cell (Chen et al., 2012). Other up-regulated DEGs in MTS0997-negative strains, *mkI*, *Rv3612c*, and *Rv3613c*, also have ESX-1-related functions, being a part of the EspR-regulated *Rv3616c-Rv3612c* operon (Raghavan et al., 2008).

Finally, *fad11*, *fadE23*, *fadE24*, and *lipQ* genes demonstrated significantly elevated expression in d_0997 and dd_0997_1338 compared to the wild type strain. All these genes are involved in *M. tuberculosis* lipid metabolism. Fad11 (fadD11) is a long-chain fatty acid-CoA ligase required for fatty acid activation, FadE23 and FadE24 are acyl-CoA dehydrogenases in cholesterol and fatty acid degradation pathways, and LipQ (Rv2485c) is a carboxylesterase/lipase. Fad11, fadE23, fadE24, and lipQ were shown to be induced during fatty acid/cholesterol breakdown within intracellular host lipid droplets, fueling long-term survival in non-replicating states (Davis et al., 2024). In addition, *lipQ* encodes highly immunogenic carboxylesterase LipQ which is of critical importance for mycobacterial virulence (Kumar et al., 2017).

To accomplish this part of work and ensure ourselves that transcriptional changes in KO strains were indeed due to the lack of MTS1338 and MTS0997 genes, we established a derivative dd_0997_1338_comp mycobacterial strain, in which deletion of genes for both sRNAs were complemented. Restoration of both MTS0997 and MTS1338 expression was confirmed by qPCR (**Supplementary Figure 5**). Also, we selected DEGs whose expression was decreased or increased in the double KO: *Rv0867*, *Rv2450* (coding for resuscitation-promoting factors RpfA and RpfE respectively), *Rv2428c*, *Rv2429c* (alkyl hydroperoxidases C and D), *Rv2485* (carboxylesterase LipQ), *Rv3614c*, *Rv3615c* and, *Rv3616c* (ESX-1 secretion-associated proteins EspD, EspC, EspA respectively). Subsequent qRT-PCR of selected DEGs confirmed that their expression in dd_0997_1338 strain complies with RNA-seq data (**Supplementary Table 2**) and significantly differs from its expression levels in dd_0997_1338_ comp. The latter was similar to that in the wild type and the control strain “complemented” with empty genetic constructs (**Figure 1F**).

### 3.3 Transcriptional profiles of infected macrophages

To evaluate potentially differential responses, which the wild type and sRNAs-deleted mycobacteria may cause in infected host phagocytes, we next used deep RNA-seq approach to assess gene expression profiles of bone marrow-derived macrophages (BMDMs) infected *in vitro* with all four mycobacterial strains. We do not consider this very common experimental approach as an optimal for modeling *in vivo* conditions (see Discussion), nevertheless, to mimic responses that occur at initial and advanced stages of infection, we infected macrophages either immediately after development of cultures, or after additional 24-h pre-activation with recombinant IFN-γ.

#### 3.3.1 Non-activated macrophages

Pairwise comparisons between cells infected with the wild type and KO mycobacterial strains revealed the following prominent differences in host transcriptional profiles.

Compared to the wild type strain, infection with the d_1338 mutant up-regulated 86 and down-regulated 80 DEGs, whereas the d_0997 mutant triggered up-regulation in only 6 DEGs, but down-regulated 143 DEGs. The double mutated dd_0997_1338 strain displayed an intermediate influence on the overall gene expression profile of the host cells, with 38 up-regulated and 60 down-regulated genes (**Supplementary Table 3**).

The most notable observation concerns a mirror-type expression profiles of genes connected with extracellular matrix (ECM) remodeling when comparing d_1338- and d_0997-infected macrophages with their wild type-infected counterparts. Infection with d_1338 strain up-regulated the expression levels of genes for collagens (Col3a1, Col4a1, Col5a1, Col5a2, Col5a3, Col6a1, Col6a2, Col6a3), laminins (Lama1, Lamb3), metallopeptidases (Adamts2, Adamts5, Adamts9, Mmp2, Mmp3), granzymes (GzmC, GzmD, GzmE), lysyl oxidases (Loxl1, Loxl2, Loxl3), and metalloproteinase inhibitors (Timp1, Timp3). Strikingly, the expression of all these genes was down-regulated after infection with d_0997. Macrophages infected with the double-knockout strain displayed the pattern of ECM-related genes expression similar to that observed after infection with the wild type strain, except up-regulated *Col5a1* and *Col5a3* genes. The results for several genes were validated using the qRT-PCR approach (**Figure 2**). Regulation of ECM remodeling aligns with a few previously reported mechanisms of mycobacterial infection pathogenesis. Thus, mycobacteria induce host proteases, e.g., MMPs, in monocyte-derived cells, neutrophils, and stromal cells. These enzymes degrade collagen of lung parenchyma, thus facilitating cavity formation in tuberculosis lesions (Squeglia et al., 2018). Granzymes, serine proteases from cytotoxic granules of innate and adaptive immune cells, further amplify tissue destruction and immune modulation in this context.

**Figure 2:**
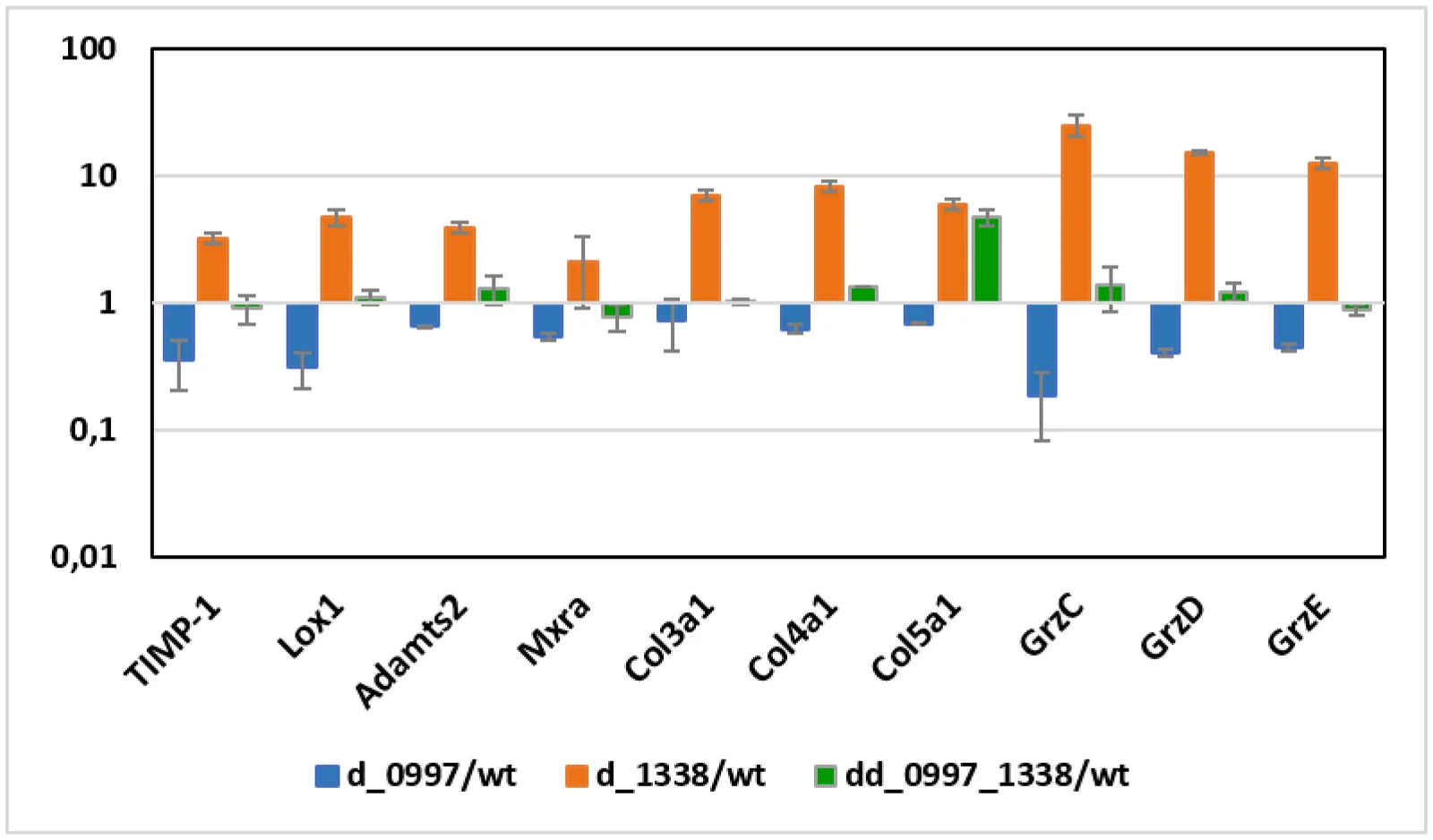
Transcriptional changes in differentially expressed genes encoding extracellular matrix proteins in nonactivated BMDMs infected with single knockout d_0997, d_1338 and double knockout dd_0997_1338 strains. Relative expression of selected DEGs encoding ECM proteins in BMDMs infected with KO and the wild type strains (MOI 2:1, 24 h p.i.). Expression levels were assessed by qRT-PCR and normalized to β-actin. Changes of expression in BMDMs infected by KO strains are shown relative to those in BMDMs infected by the wild type strain. The results from three biologically independent experiments are shown.

Apart from genes involved in ECM dynamics, d_1338 infection up-regulated genes associated with mitotic entry and cell cycle progression, including *Cdca3*, *Cdca8*, cyclin-dependent kinases (*Cdk1*, *Cdk6*, *Cdk12*), and their inhibitors (*Cdkn2c*, *Cdkn2d*, *Cdkn3*) (**Table 1**). This gene signature is consistent with interference of the KO strain with host cell proliferation.

**Table 1:** The biological pathways repressed in d_1338-infected non-activated BMDMs.

Pathway	Genes	Fold enrichment	FDR
GO:0032727 Positive regulation of interferon-alpha production	*Dhx36, Tlr9, Ifih1, Dhx9*	39,8	5,21E-04
GO:0032647 Regulation of interferon-alpha production		33,3	9,06E-04
GO:0002218 Activation of innate immune response	*Tlr9, Mndal, Trim30d, Matr3, Ifi207, Ifi203*	25,9	3,64E-05
GO:0051059 I-kappaB/NF-kappaB signaling	*Tlr9, Ash1l, Rel, Card11, Ednrb, Usp25*	22,1	7,26E-05
GO:0032728 Regulation of interferon-beta production	*Dhx36, Tlr9, Ifih1, Dhx9*	15,4	9,78E-03
GO:1905269 Regulation of chromatin organization	*Jarid2, Ash1l, Ercc6, Ifi207, Ep300, Tbl1xr1, Kmt5b, Chek1, Rb1*	9,8	2,93E-02

Non-activated BMDMs were infected with the d_1338 KO strain or the corresponding wild type control strain (MOI 2:1). Total RNA was isolated 24 h post-infection and subjected to RNA sequencing to characterize genome-wide gene-expression profiles. Downregulated differentially expressed genes were significantly enriched in pathways associated with type I interferon signaling.

Conversely, the pathways repressed in d_1338-infected cells included type I interferon (IFN-I) synthesis and IFN-I-responsive biochemical cascades (**Table 1**). Among repressed are genes encoding the following helicases and sensors, suppression of which is consistent with dampening innate immunity: DHX9 (DEAH-box helicase involved in RISC complex assembly via siRNA recognition (Fu and Yuan, 2013); DDX60 (helicase activating RIG-I and MDA5 for IFN-I production (Oshiumi et al., 2015); DHX36 (part of the DDX1-DDX21-DHX36 RNA sensor complex enhancing RIG-I signaling (Zhang et al., 2011)); DDX46 (DEAD/DEAH-box helicase negatively regulating antiviral responses through mRNA nuclear retention (Zheng et al., 2017)); MDA5 (sensor of long dsRNA triggering innate immunity), and TLR9 (endosomal DNA sensor activating NF-κB via MyD88 (Huang and Yang, 2010)).

After infection of macrophages with dd_0997_1338 strain, we observed that the group of up-regulated DEGs is strongly enriched for genes participating in immune activation pathways, innate immunity, antigen presentation and inflammation, i.e., the hallmarks of macrophage activation under infectious stress (**Supplementary Table 3**). This transcriptional profile is characterized by up-regulation of genes for the markers of pro-inflammatory monocytes (Ly6c1, Ly6c2, Ly6i, Mcemp1), macrophage/T-cell recruiting chemokines (Ccl5/RANTES), antimicrobial peptides (Camp/cathelicidin), MHC class II molecules (H2-Aa, H2-Ab1, H2-Eb1 requisite for CD4+ T-cell priming), B-cell factors (Tnfsf13), anti-apoptotic regulators (Bcl2a1d, G0s2, Ifitm1), and ECM components (Col5a1, Col5a3, Thbs2, Cst6/7). Down-regulated pathways encompassed IFN-I signaling (Mx2, Ulbp, Mysm1), hypoxia response (HIF-2α), and chromatin remodeling (Atrx for heterochromatin maintenance, Arid4b via RB pathways, Top2b for DNA resolution). These shifts may reflect adaptive cellular responses to infection-induced stress, genomic instability and epigenetic control.

#### 3.3.2 IFN-γ activated macrophages

Most prominent shifts in gene expressed profile were observed in BMDMs infected with the dd_0997_1338 double KO strain (91 genes up-regulated, 100 genes down-regulated), whereas infection with d_1338 and d_0997 single-knockout strains resulted in 14/18 and 8/13 up-regulated/down-regulated genes, respectively (**Supplementary Table 4**). Since alterations in transcriptional response to infection were substantially more pronounced in double-knockout infected cells, we focused our functional analyses on dd_0997_1338-infected group (**Supplementary Table 4**). Functional enrichment analysis of changes caused by dd_0997_1338-triggered infection revealed overrepresentation of DEGs reflecting imbalances in immune responses (**Figures 3A, B**; **Table 2**). Thus, up-regulation of biological pathways GO:0006955 (immune response) GO:0002253 (activation of immune response), driven by genes encoding Tlr9, C3ar1, Cx3cr1, Nlrp1b, is characteristic for active TB. The up-regulated pathways GO:0035458 (cellular response to interferon-beta) and GO:0035456 (type I IFN-related signatures), which are represented by the genes for Ifi209, Ifi208, Ifi213, and Oas1c, are known to be increased in TB lesions and blood, particularly during active phases of the disease, neutrophil influx and active mycobacterial replication (Akter et al., 2022; Kotov et al., 2023). Genes *Vsir*, *Il2ra*, and *Card11* are associated with pathways regulating CD4+ T cell proliferation (GO:2000561), where Il2ra and Card11 promote expansion critical for IFN-γ production and TB bacterial control (Carter and Pomerantz, 2022; Ross and Cantrell, 2018), while Vsir (V-domain Ig suppressor of T cell activation) inhibits it (Lines et al., 2014). Such a functional imbalance might contribute to the T-cell exhaustion during chronic TB (Lombardi et al., 2021; Pan et al., 2023).

**Figure 3:**
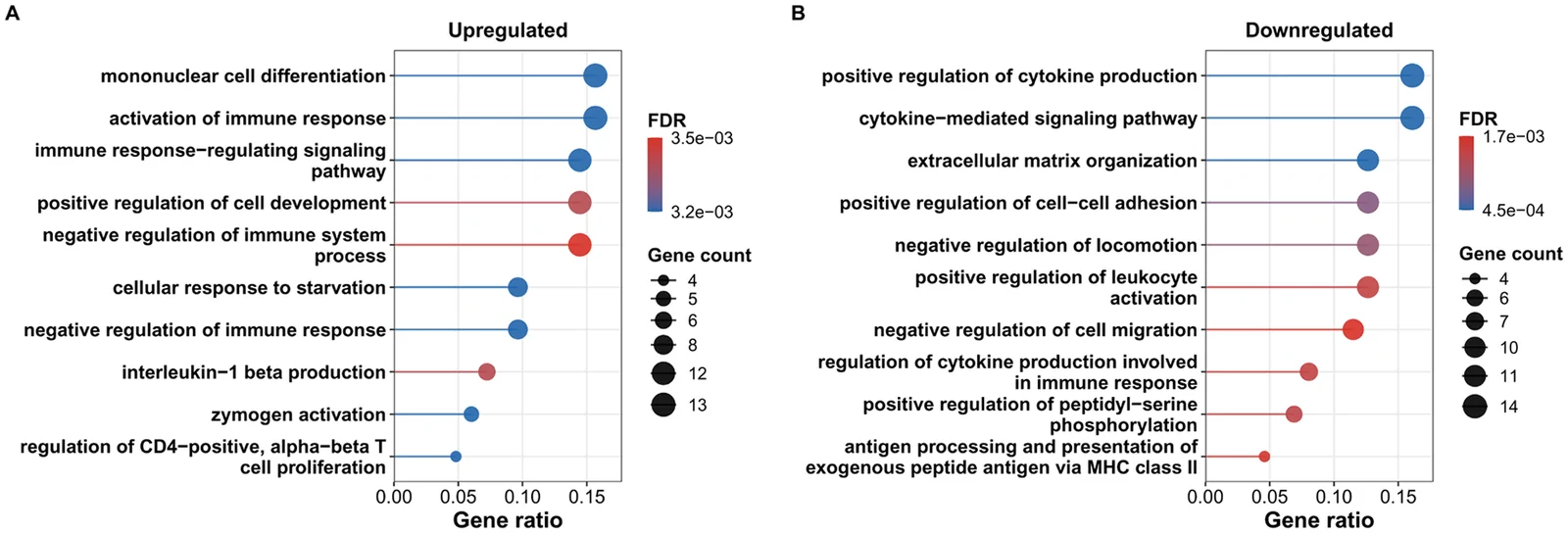
Functional enrichment analysis of DEGs in INF-γ-activated BMDMs infected with the double KO and wild type strains. Lollipop plots show significantly enriched metabolic pathways identified by Gene Ontology analysis. The size of each bubble indicates the number of genes associated with the respective pathway, whereas color intensity represents statistical significance based on the false discovery rate (FDR). **(A)** upregulated DEGs; **(B)** downregulated DEGs.

**Table 2:** The biological pathways enriched in IFN-γ stimulated BMDMs infected by dd_0997_1338 strain.

Pathway	Genes	Fold enrichment	FDR
Up-regulation			
GO:2000561 reg. of CD4-positive alpha-beta T cell proliferation	*Vsir Il2ra Card11 Cd55*	46,0	0,000395
GO:0035458 cellular response to interferon-beta	*Ifi209 Gm5431 Ifi208 9930111J21Rik2 Ifi213 Oas1c*	27,9	2,81E-05
GO:0035456 response to interferon-beta	*Ifi209 Gm5431 Ifi208 9930111J21Rik2 Ifi213 Oas1c*	24,1	5,86E-05
GO:0002253 activation of immune response	*Cd55 C3ar1 Tlr9 Fcrl5 Nfatc2 Card11 Ifi209 Ifi208 Ifi213 Mef2c Cd5l*	9,5	9,48E-06
GO:0002684 positive reg. of immune system proc.	*Vsir Cd55 C3ar1 Evi2 Gm5150 Evi2b Fos Gfi1b Tlr9 Fcrl5 Il2ra Nfatc2 Card11 Ifi209 Cx3cr1 Ifi208 Ifi213 Mef2c Cd5l Pecam1 Ccl12*	6,2	1,62E-08
GO:0050776 reg. of immune response	*Cd55 C3ar1 Tlr9 Nlrp1b Vsir Fcrl5 Nfatc2 Card11 Ifi209 Ifi208 Ifi213 Mef2c Cd5l Fgl2 Oas1c*	4,9	9,43E-05
GO:0002682 reg. of immune system proc.	*Vsir Cd55 Gfi1b C3ar1 Tlr9 Evi2 Gm5150 Evi2b Nlrp1b Fos Fcrl5 Il2ra Nfatc2 Card11 Ifi209 Cx3cr1 Ifi208 Ifi213 Mef2c Cd5l Gcnt1 Fgl2 Oas1c Pecam1 Ccl12*	4,6	3,64E-08
GO:0006955 immune response	*Cd55 Ccl12 Ccr3 Trim14 C3ar1 Tlr9 Cx3cr1 Nlrp1b Vsir Fcrl5 Ltb Il2ra Nfatc2 Card11 Tnfsf10 Mfsd6 Ifi209 Ifi208 Ifi213 Mef2c Cd5l Lfng Fgl2 Oas1c*	4,0	1,26E-06
Down-regulation			
GO:0034341 response to interferon-gamma	*Ccl20 Ubd Gbp5 Nos2 Ciita Cd74 H2-Aa H2-Ab1 Wnt5a Tnf*	16,7	2,19E-07
GO:0034097 response to cytokine	*Timp1 Tnf Cd74 Ccl20 Cxcl1 Il13ra2 Ubd Cxcl2 Gbp5 Nos2 Tnfsf11 Ciita H2-Aa H2-Ab1 Aldh1a2 Wnt5a Osmr Il1rl1 Ereg Ccrl2 Spry4 Cav1*	5,8	1,80E-08
GO:0016477 cell migration	*Ccl20 Cxcl1 Ccrl2 Lamb2 Cxcl2 Tnf Cd74 Krt16 Timp1 Cav1 Twist2 Dcn Wnt5a Tnfsf11 Ednrb Gdnf Adamts1 Igfbp5 Nr4a3 Plpp3 Slamf1 Plk2 Adamts9 Mmp10 Syne2 Adora2b*	4,3	6,54E-08
GO:0002376 immune system proc.	*Cd74 Ccl20 Ptx3 Cxcl1 Ubd H2-Aa Ccrl2 Il27 Cxcl2 H2-Ab1 Gbp5 Slamf1 Nos2 Wnt5a Tnfsf11 Ciita Tnf Cav1 Arg2 Ednrb Lif Tnfsf15 Krt16 H2-Eb1 Il1rl1 Adora2b Satb1 Fam20c Nr4a3 Ereg Il13ra2 Hcar2*	3,1	4,72E-07
GO:0009605 response to external stimulus	*Nos2 Ccl20 Ptx3 Cxcl1 Ubd Ccrl2 Cxcl2 Gbp5 Cd74 Adora2b Wnt5a Tnfsf11 Ciita Tnf H2-Aa Gdf15 Il27 H2-Ab1 Cav1 Aldh1a2 Slamf1 Arg2 Ednrb Krt16 Adamts9 AA467197 Gdnf Adamts5 Lamb2 Satb1 Clic5 Il1rl1 Nr4a3 Ereg Bhlhe40 Angptl4*	3,2	2,84E-08

BMDMs were preactivated for 24 h with recombinant IFN-γ (100 U/mL) and subsequently infected with either the dd_0997_1338 KO strain or the corresponding wild type control strain (MOI 2:1). Total RNA was isolated 24 h post-infection and subjected to RNA sequencing to characterize genome-wide gene-expression profiles. Functional enrichment analysis of the transcriptional changes induced by infection with the dd_0997_1338 KO strain revealed an overrepresentation of DEGs associated with dysregulated immune responses.

The list of down-regulated DEGs also shows important differences between macrophages infected with KO and wild type mycobacterial strains. Thus, compared to the wild type strain, infection with dd_0997_1338 resulted in strong down regulation of genes for key regulators of antigen presentation (Cd74, Ciita, H2-Aa, H2-Ab1, H2-Eb1), innate immune activation (Nos2, Gbp5), chemokine production (Cxcl1, Cxcl2, Ccl20), and extracellular matrix remodeling (Mmp3, Mmp10, Mmp13, Adamts1, Adamts5, Adamts9 and Timp1). Decreased expression of genes for molecules involved in cytokine signaling (TNF, IL-11, IL-27, Osmr), stress adaptation (Adora2b, Pim3, Ppp1r15a, Gdf15), and stromal responses (Wnt5a and Igfbp5) is suggestive for a broad suppression and/or reprogramming of immune/inflammatory pathways in phagocytes infected with mutated bacteria.

Only three annotated DEGs, two of which are protein-coding genes, appeared in all comparisons, all down-regulated following infection with KO strains. In the context of present study, remarkable are *Nos2* encoding inducible nitric oxide synthase critical for NO-mediated mycobacterial killing in macrophages (MacMicking et al., 1997), and the gene for 9930111J21Rik2, a member of murine immune reactive GTPases family (IRGs) involved in intracellular pathogen protection (Taylor et al., 2007).

### 3.4 sRNAs deletions enhance mycobacterial virulence *in vivo*

To test whether shifts in gene expression profiles after sRNAs deletions alter virulence of corresponding *M. tuberculosis* strains, we next compared survival curves of mice infected with mycobacteria under study. For these and other experiments described below we used genetically TB-susceptible mice of the I/St strain (Nikonenko et al., 2000), which allows for accomplishing survival experiments within reasonable time limits (< 1 year). Mice were identically challenged with moderate doses of mycobacteria (~100 CFU per mouse) of all four strains under study administered via respiratory tract using aerosol chamber. Kaplan-Meier analysis for multiple comparisons demonstrated that the lifespan of mice infected with all three KO strains was equally and significantly shorter than that of mice infected with the wild type strain (**Figure 4A**).

**Figure 4:**
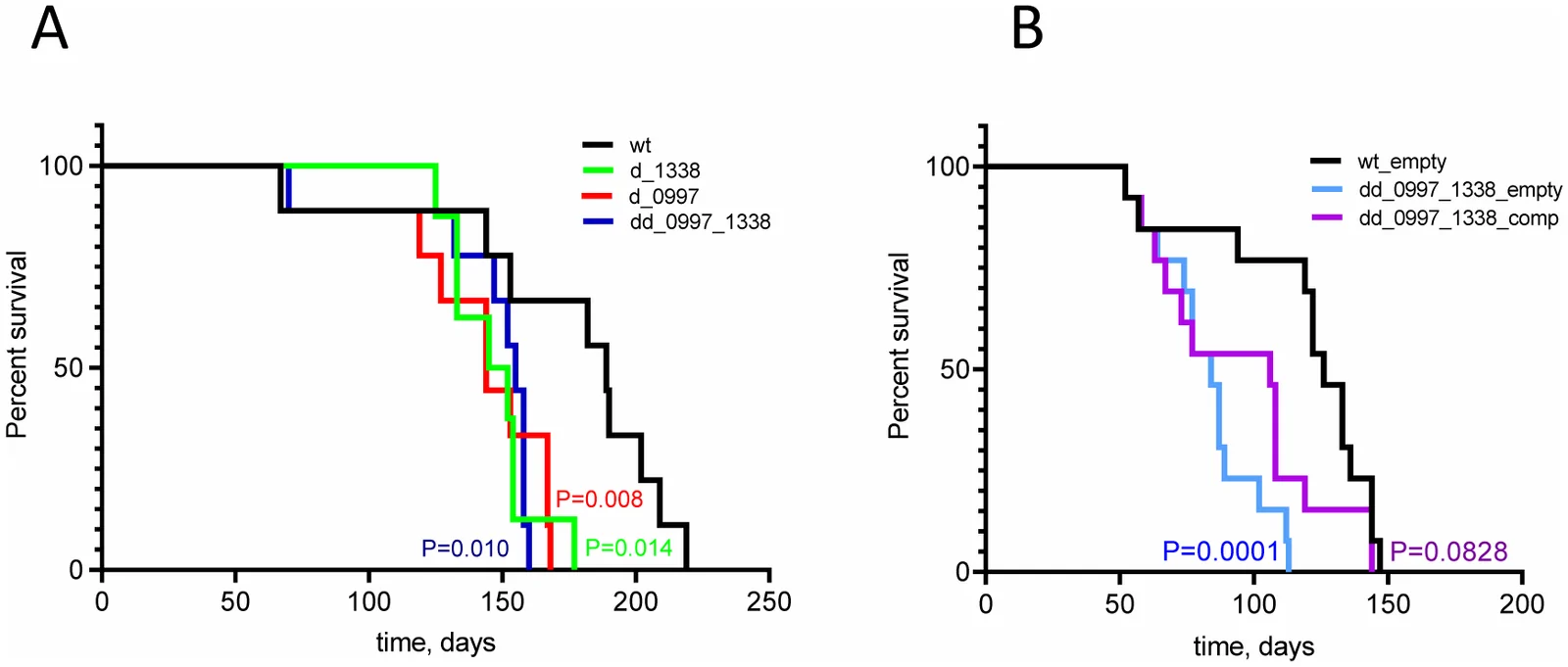
Survival of mice infected with the KO and the wild type strains. **(A)** Aerosol infection (low-dose, ~100 CFU per mouse, 9 mice per each group) of TB-susceptible I/St mice with the KO mutants and the wild type strains. **(B)** Intravenous infection (high dose, ~10^6^ CFU per mouse, 13 mice per each group), of TB-susceptible I/St mice with the double-KO and the wild type strains transformed with the empty pMV306_rrnB vector (dd_0997_1338_empty and wt_empty, respectively) and complemented double-KO strain (dd_0997_1338_comp). Survival was monitored daily starting at day 50 post-challenge; survival curves were compared using the log-rank (Mantel-Cox) test.

Here, we should note that these single-knockout mutants (d_0997 and d_1338) were previously evaluated for virulence in a murine infection model (Shepelkova et al., 2021). The survival outcomes reported in that study differ from those observed here. These discrepancies are likely attributable to differences in experimental design: Shepelkova et al. employed intravenous administration of a high dose (~10^6^ CFU), whereas our study used a low-dose aerosol challenge (~100 CFU), a route and dose that more closely mimic natural infection and the ensuing host immune response.

To confirm directly that the accelerated mortality phenotype is due to sRNAs deletion, we compared the complemented strain dd_0997_1338_comp with the wild type strain (the pseudo-complemented strain carrying an empty pMV306_rrnB vector). Since the double-knockout strain displayed the strongest level of virulence *in vivo* and induced the most prominent transcriptome shifts in IFN-γ activated macrophages, it was selected for complementation studies, for which we applied a high-dose intravenous challenge to shorten the duration of experiments. The group infected with the complemented strain survived significantly longer than that infected with the dd_0997_1338 strain and did not differ statistically from the wild type control, although complementation seemingly was only partial (**Figure 4B**). Nevertheless, Kaplan-Meier analysis confirmed statistical differences between mice infected with the dd_0997_1338_comp and dd_0997_1338 strains (P< 0.01). Thus, complementation for deleted *MTS1338* and *MTS0997* genes resulted in restoration of the “low virulence” phenotype, indicating the involvement of mycobacterial sRNAs in regulation of host-pathogen interactions during experimental TB.

We next assessed possible influence of the sRNAs presence on mycobacterial multiplication in the lungs of infected mice. Enumeration of mycobacterial lung CFU counts at week 6 post-challenge demonstrated no statistical differences between groups (**Figure 5A**). Thus, our observation provided yet another example of inconsistency between CFU counts and mortality data. Observations decoupling lung mycobacterial burden from pathological outcomes in different experimental settings are numerous and were recently discussed in considerable detail (Gern et al., 2025; Jacobs, 2026).

**Figure 5:**
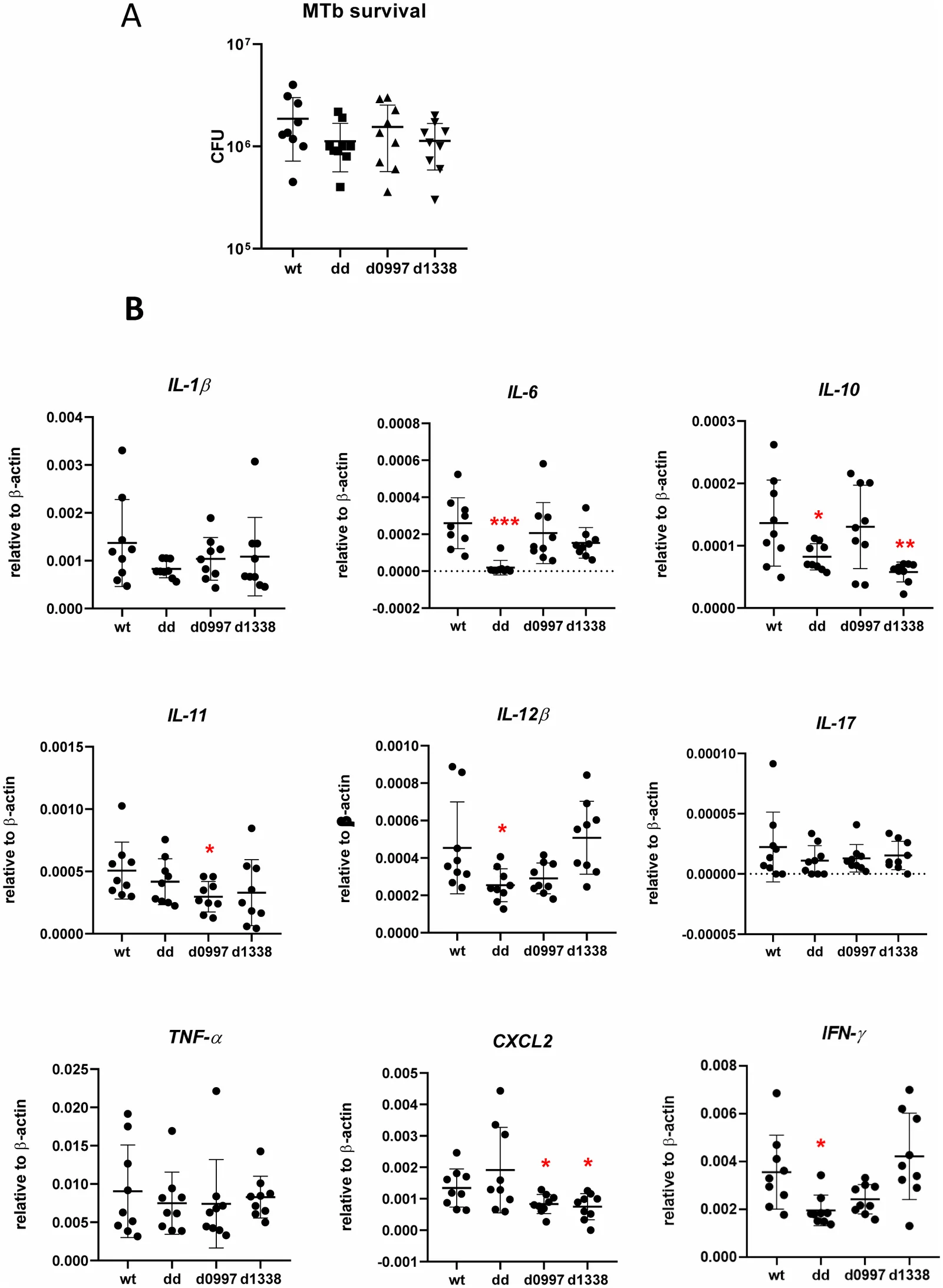
Lung infection with the KO and the wild type strains. **(A)** CFU numbers in the lungs of mice infected with the KO and the wild type strains. (9 mice per each strain, low-dose aerosol challenge, ~100 CFU per mouse, 6 months p.i.). The number of CFU is given for two right lobes of the lung of each mouse (see Materials and Methods for details). **(B)** Transcription of pro- and anti-inflammatory genes in lungs of mice infected with the KO and the wild type strains. Transcription levels were determined by qRT-PCR and normalized to that of β-actin mRNA. The data are presented as the mean ± SD of nine biological replicates for each strain; *p < 0.05, **p < 0.01, ***p<0.001.

### 3.5 Immune responses in infected lungs

#### 3.5.1 Cytokine gene expression

To characterize further *in vivo* parameters of host response to infection induced by *M. tuberculosis* strains, we applied two different approaches. First, we assessed the expression level of genes for key pro- and anti-inflammatory cytokines in the lung tissue of infected mice using qRT-PCR. Second, we quantified lung immune cell populations in infected animals by flow cytometry.

We found no differences in the expression levels of genes for IL-1b, IL-17 and TNF in lungs of mice infected with mycobacterial strains under study (**Figure 5B**). However, the expression level of genes for pro-inflammatory IL-6, IL-12b and IFN-γ was significantly lower in the group infected with the dd_0997_1338 double-KO strain, the gene for IL-11 was down-regulated in mice infected with the d_0997 strain, the gene for CXCL2 – in mice infected with d_0997 and d_1338 strains, whilst the gene for anti-inflammatory IL-10 – in mice infected with d_1338 and dd_0997_1338 strains. Lower level of transcription of pro-inflammatory *il6*, *il12b*, and *infg* may reflect a shift in the host-pathogen balance from “immune protection” to “bacterial persistence”, preventing formation of effective granulomas and facilitating transition to progressive TB infection.

#### 3.5.2 Immune cell populations

The total numbers of cells in individual samples of lung tissue digests were similar and did not differ between animals infected with the wild type and KO *M. tuberculosis* strains (16–22 x 10^6^, non-significant). Enumeration of major immune cell populations by flow cytometry demonstrated the lack of differences in the content of CD8+ T-cells, Ly-6G+ neutrophils, as well as CD11b+CD11c−Ly6G− and CD11c+ phagocytes between groups infected with four mycobacterial strains under study (**Figure 6A**). By contrast, the amount and frequency of CD4+ T-cells gated for either total cellularity or for lymphocytes was lower in mice infected with dd_0997_1338 strain compared to control animals (**Figure 6B**). We also detected a decrease in B-cell frequency in lungs of mice infected with both single KO d_0997 and d_1338 strains but, to our surprise, not with double KO strain. Analogously, infection with either d_0997 or d_1338 strain resulted in a 2-fold increase in the expression of the MHC-II H2-A molecules on the surface of antigen-presenting cells (APC); however, mice infected with the double KO mutant did not differ from controls with this regard. The latter phenotype was not due to B-cell deficiency in dd_0997_1338-infected mice since Class-II staining was performed separately for different populations of APC (**Figure 6B**).

**Figure 6:**
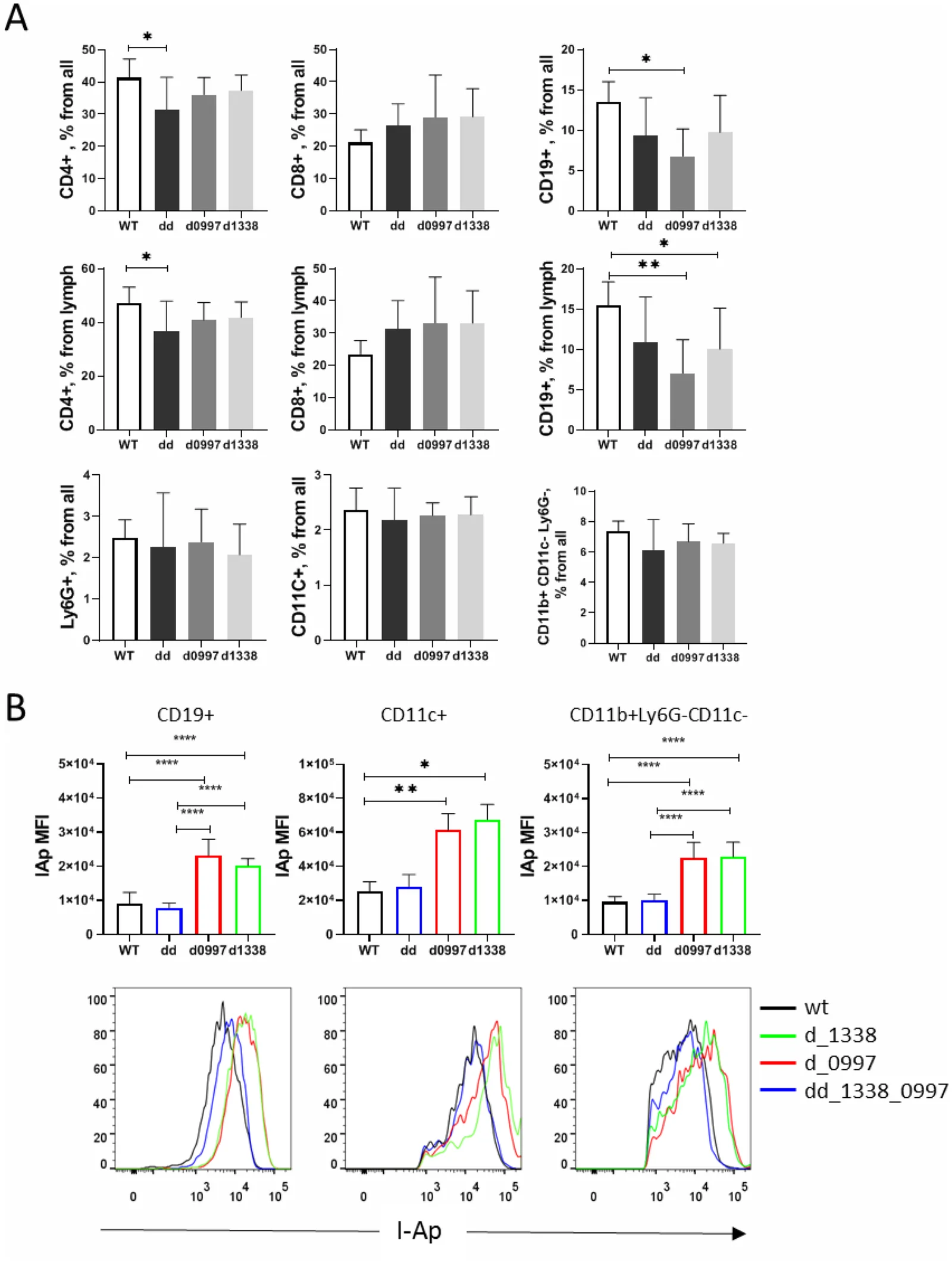
Flow cytometry analysis of immune cell populations in the lungs of mice infected with the KO and the wild type strains (9 mice per each strain, low-dose aerosol challenge, ~100 CFU per mouse, 6 months p.i.). **(A)** Frequency of CD4^+^ and CD8^+^ T cells and CD19^+^ B cells in all lung cells (upper panels) and in lymphocytes (middle panels); frequency of Ly6G^+^ neutrophils, and CD11c^+^ and CD11b^+^Ly6G^-^CD11c^-^ myeloid cells (lower panels). **(B)** Expression of MHC-II (I-Ap) on professional antigen-presenting cells (CD19+ B cells and CD11c^+^ and CD11b^+^Ly6G^-^CD11c^-^ phagocytes) is shown as the mean of fluorescence intensity (MFI). The results were analyzed with one-way ANOVA; *p < 0.05, **p < 0.01, and ****p < 0,0001.

## 4 Discussion

The essence of our findings is a direct demonstration that switching off particular regulatory sRNAs in the genome of *M. tuberculosis* results not only in multiple shifts in its gene expression profile and virulence, but prominently changes immune response pathways in infected cultured macrophages and lungs of infected murine host. RNA-seq analysis of the d_0997, d_1338 and dd_0997_1338 mid-log phase cultures revealed that d_0997 and double KO dd_0997_1338 mutants display very similar transcriptomes, which differ significantly from that of the wild type strain. At the same time, d_1338 mutant displays only minimal transcriptional changes.

Previous search for targets of these two sRNAs provided somewhat ambiguous results. MTS0997 was shown to up-regulate *Rv3282*, *fadA3*, and *lipQ* genes required for fatty acid production in mycobacteria by binding their upstream regions (Girardin and McDonough, 2020). However, we did not detect these genes among DEGs in d_0997 strain. This discrepancy may be explained by different experimental settings of the two studies: in the work cited above, MTS0997 targets were reported for fatty acid-deficient conditions. Regarding MTS1338, despite considerable efforts others and we were unable to identify any reliable target. Nevertheless, we previously demonstrated that MTS1338 is secreted into the cytoplasm of infected macrophages (Bychenko et al., 2023). Thus, it cannot be excluded that MTS1338 targets rather eukaryotic than bacterial products, and the absence of DEGs in d_13398 transcripts indirectly supports this hypothesis. Of note, although under cultural conditions d_1338 transcriptome is very similar to that of the wild type parental strain, it differs significantly in non-activated BMDMs. We hypothesize that after MTS1338 secretion into cytoplasm of infected macrophage, bacterial sRNA may bind the host RNA sensors RIG-I or MDA5. As mentioned above, transcriptional changes after d_1338 infection include several protein-coding genes (*Dhx36*, *Tlr9*, *Ifih1*, *Dhx9*) related to positive regulation of IFN-α and IFN-β production. No similar changes were found in macrophages infected with d_0997 or double mutant strain.

Comparative analysis of transcriptomes demonstrated that the double-knockout strain elicited the most prominent and functionally relevant response in macrophages, particularly in IFN-γ- activated. Hence, we mostly used this strain for long-lasting and costly *in vivo* experiments; however, the key survival and lung CFU count phenotypes included also single KO strains. Such a combination allowed for examining combined effects of MTS0997 + MTS1338 deletions, their possible genetic interactions and individual contribution of two sRNAs in TB progression.

An important issue arising from our *in vivo* experiments is a discrepancy between survival time and lung bacterial burden phenotypes. Although mice infected with single and double-knockout mycobacterial strains survived significantly shorter than those infected with the wild type strain, lung CFU counts at 6 weeks post infection did not differ between the groups. These results do not necessarily mean that deletions of sRNA under study failed to affect mycobacterial virulence. The term “virulence” primarily refers to severity and progression of a disease, rather than to the rate of pathogen’s multiplication. This is particularly true for mycobacterial and other intracellular chronic bacterial infections, in which host mortality results predominantly from tissue damage and dysregulated immune inflammation rather than from direct effects of bacterial replication. Dissociations between mycobacterial growth, lung tissue damage and host mortality are amply described for both experimental TB models and clinical settings (reviewed in Gern et al., 2025; Jacobs, 2026). In this context, lung CFU counts and survival curves represent very distant readouts. CFU enumeration reflects the bacterial burden at a defined time point, whereas survival integrates the cumulative effects of bacterial factors, tissue pathology, and host inflammatory responses over the course of disease.

The most challenging part of all investigations combining microbiological and immunological approaches for the studying infectious diseases is co-interpretation of the results obtained *in vitro* and *in vivo*. Ours is not an exemption, which is well illustrated by opposite tendencies in regulation of expression of genes for MHC-II in infected BMDM and in the lung tissue of TB-infected mice. A decreased expression of the *h2aα*, *h2aβ* and *h2eb* MHC-II genes, as well as their master regulator *ciita*, in macrophages infected with dd_0997_1338 double KO strain (**Supplementary Table 3**) is consistent with down-regulation of MHC class II-dependent antigen processing and suboptimal CD4+ T-cell activation. However, the results of flow cytometry of lung cells from infected mice (**Figure 6C**) clearly demonstrate that under conditions of real disease progression, and measured at the protein level, MHC-II expression is significantly elevated when the host collides with more virulent pathogen variants. Although genetic differences between the sources of host cells analyzed in the two experimental settings might account for this discrepancy, we consider this not very likely. In mice, whose high level of TB susceptibility depends on MHC-II, not its expression level but suboptimal CD4 T-cell – neutrophil interactions are primary cause of TB sensitivity (Logunova et al., 2024, 2025). A more likely explanation is that in an infected macrophage cultures we observe the outcome of a successful parasite’s attempt to block an essential part of adaptive protective immunity (non-existing under the given experimental conditions), whereas in infected organ, where all components of immunity are available, the host reverses the situation, at least temporary.

Similarly, physiological consequences of lower expression of genes for powerful attractants of neutrophils, CXCL1 and CXCL2, after infection of INF-γ-activated BMDM with dd_0997_1338 (**Supplementary Table 4**, **Table 2**) was not reflected *in vivo* by a decrease in neutrophil lung infiltration (**Figure 6B**). Thus, a good care should be taken when translating the results of host gene profiling in *in vitro* infection models onto the pathways of immune response and inflammation *in vivo*. In addition, transcriptional epistasis, a poorly investigated phenomenon very recently reported in a study of host TB genetics using single- and double-KO mouse strains (Dallmann-Sauer et al., 2026), may well operate in parasite genetics as well, hampering interpretations. Regarding this study, B-cell and Class-II expression deficiencies in lungs of mice infected with single mycobacterial KO strains and the absence of these phenotypes in double KO-infected animals (**Figures 6A, B**) may serve as examples of such interactions.

It should also be emphasized that *in vivo* observations reported herein were made in mice kept under non-SPF and non-sterile conditions. Experimentation in SPF animals would most probably provide more consistent results with smaller deviations; however, disease and immune response phenotypes observed in ‘dirty mice’ may be more relevant to general human populations, as was discussed in considerable detail during the NIH-supported meeting (https://cvent.me/4YenkK).

## Data Availability

The datasets presented in this study can be found in online repositories. The names of the repository/repositories and accession number(s) can be found in the article/**Supplementary Material**.
