# Supplementary material for: Mycobacterium tuberculosis small RNAs MTS0997 and MTS1338 regulate both pathogen virulence and host immune responses
Source: Front Cell Infect Microbiol. 2026 Sep 28;16:1918881. doi: 10.3389/fcimb.2026.1918881 (PMC13654791; doi:10.3389/fcimb.2026.1918881)
Supplement: Supplementary file 1 [file DataSheet1.pdf]

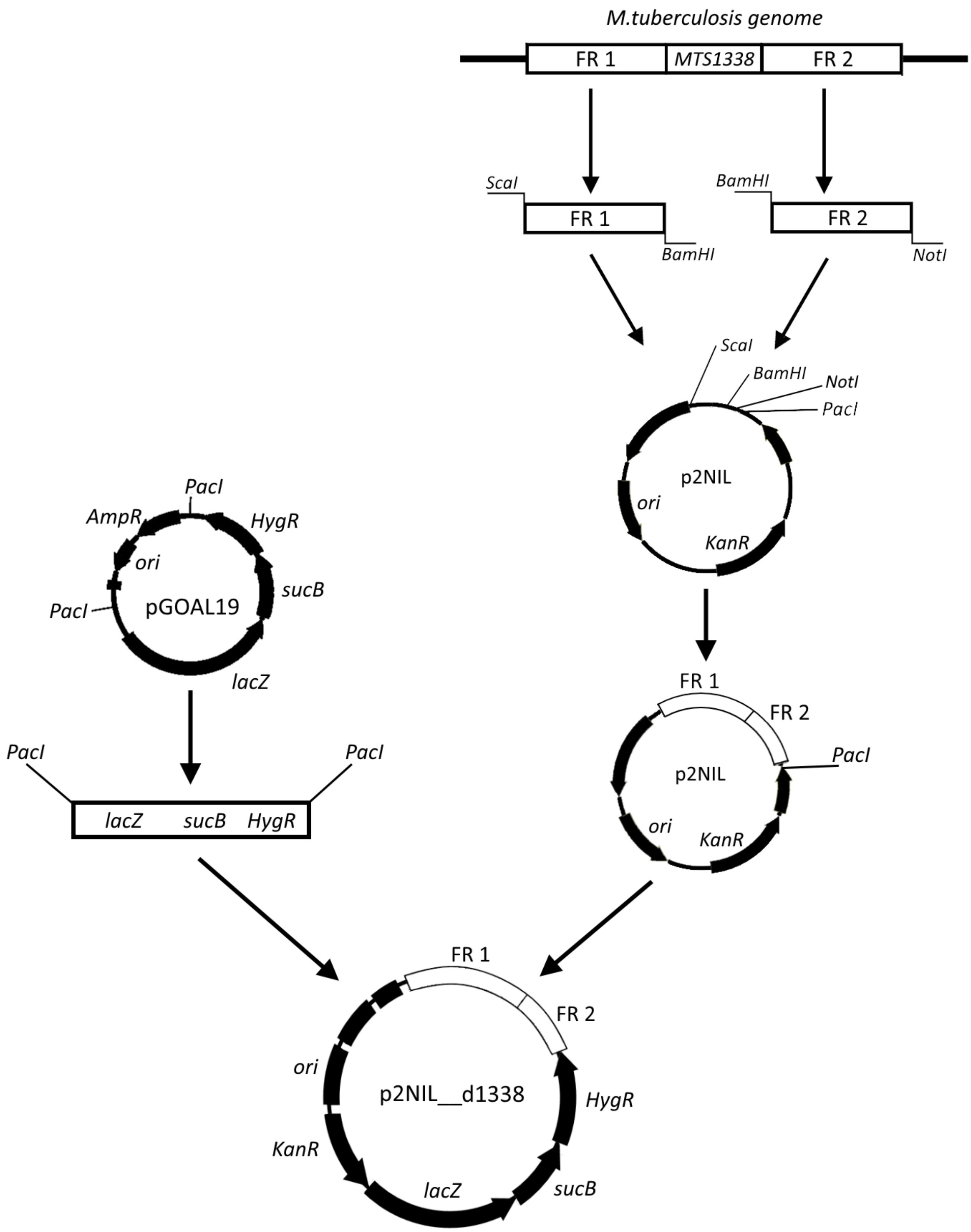

Supplementary Figure 1. Scheme of construction of KO suicide vector p2NIL_d1338. p2NIL_d0997 was constructed the same way. (Figure was modified from Parish and Stoker, 2000).

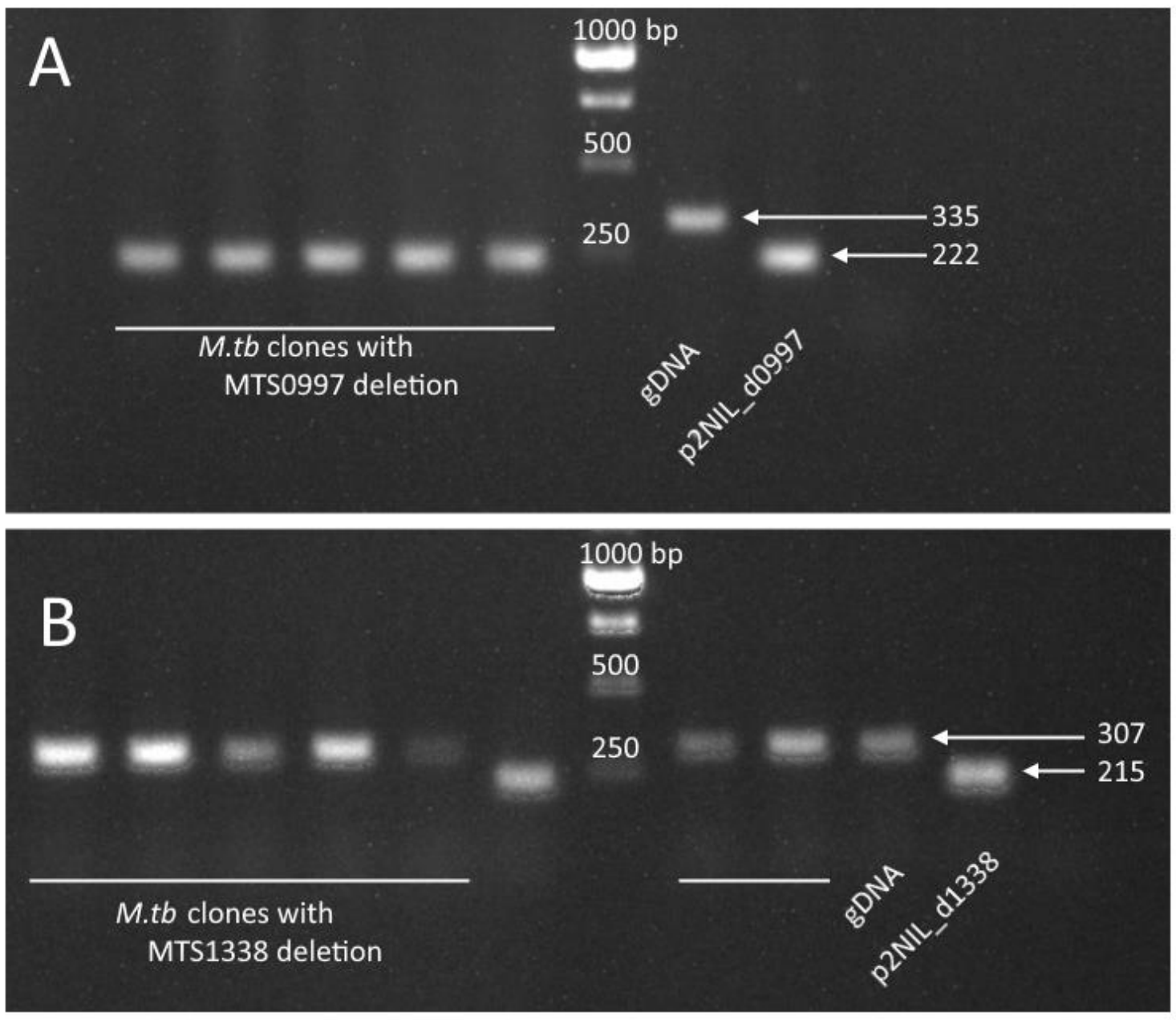

Supplementary Figure 2. PCR screening of the obtained target clones d_0997 (A) and d_1338 (B). In the case of gene deletion, a smaller PCR product size is visible on gel compared to strains that retained the wt genotype and the small RNA gene.

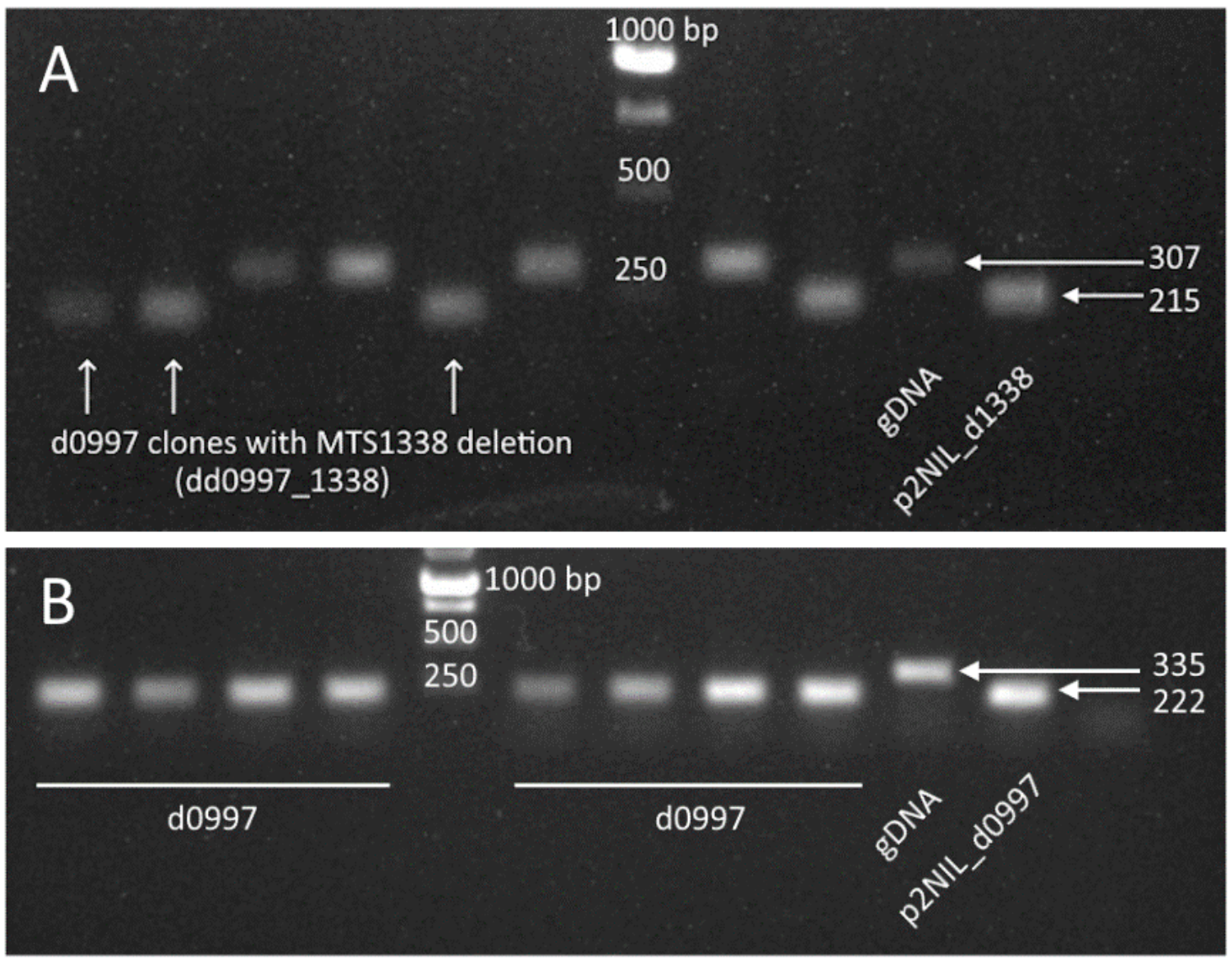

Supplementary Figure 3. PCR screening of the obtained dd_0997_1338 target clones for the MTS1338 gene deletion in d_0997 strain. A smaller PCR product size corresponds to the deletion genotype, while a larger one indicates retention of the MTS1338 gene (A). Confirmation of the MTS0997 gene initial deletion in clones with dd_0997_1338 (B).

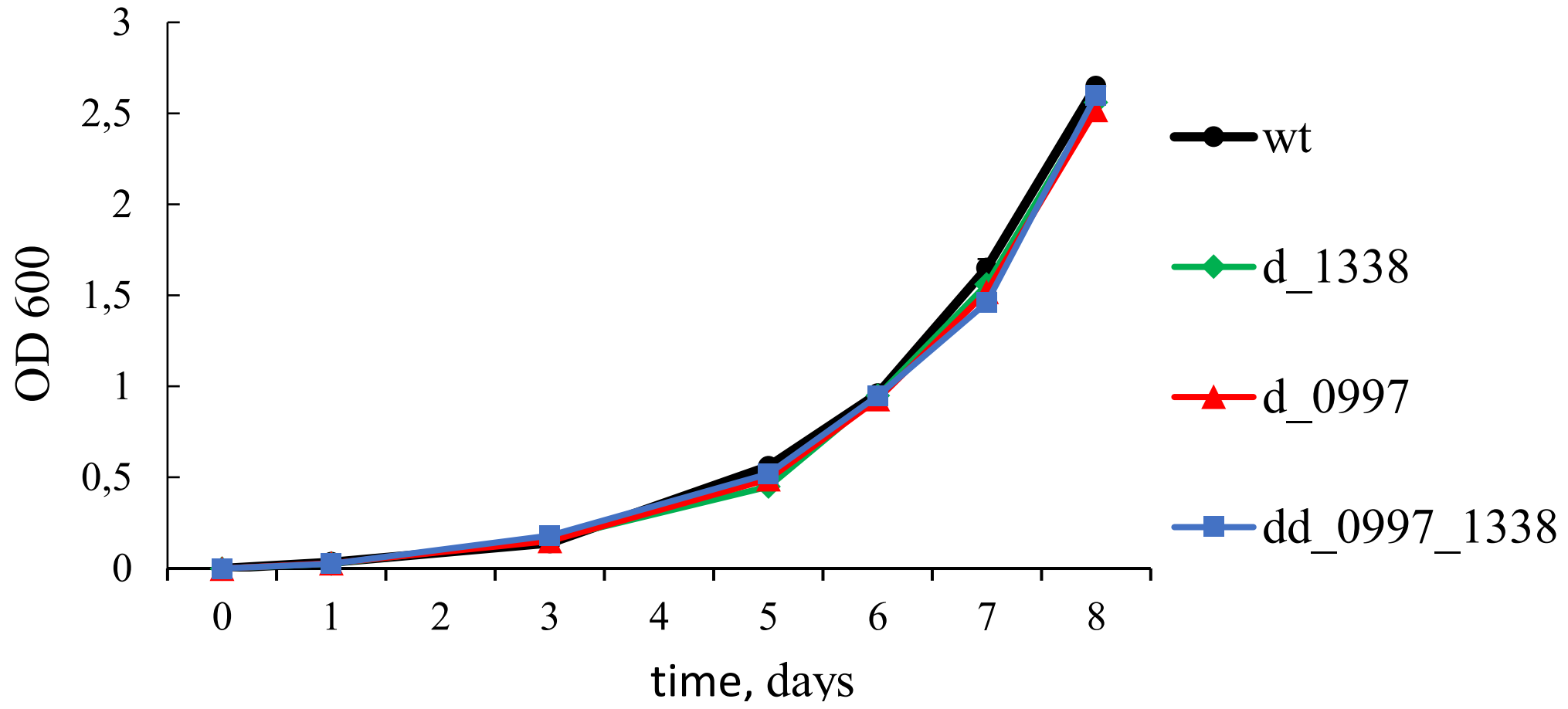

Supplementary Figure 4. Growth dynamics of wt, d_0997, d_1338 and dd_0997_1338 strains of *M. tuberculosis* in Middlebrook 7H9 medium supplemented with 10% ADC and 0,05% Tween-80.

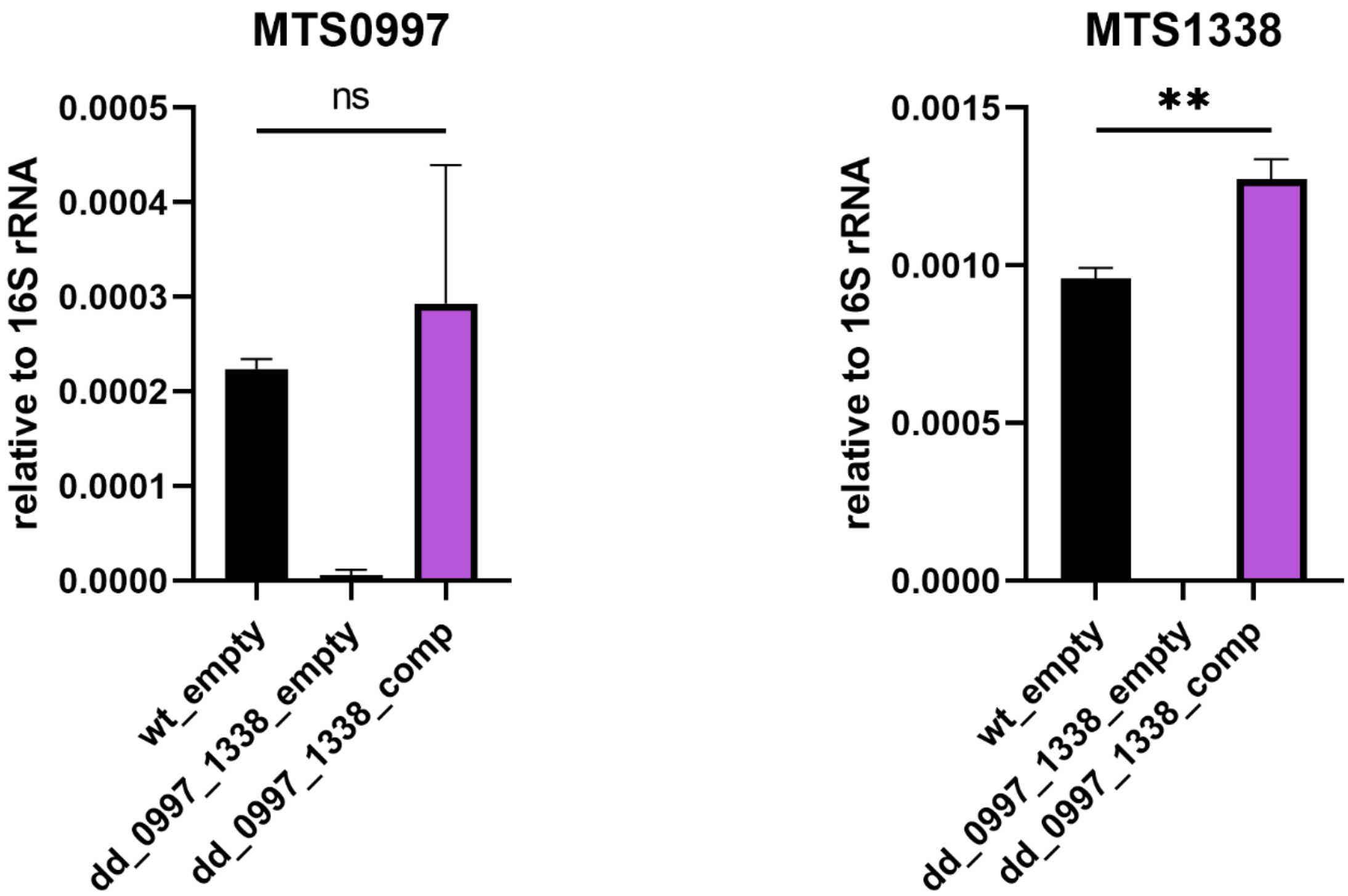

Supplementary Figure 5. MTS1338 and MTS0997 expression in dd_0997_1338_comp confirmed by the qPCR.
