# Supplementary material for: Mycobacterium tuberculosis small RNAs MTS0997 and MTS1338 regulate both pathogen virulence and host immune responses
Source: Front Cell Infect Microbiol. 2026 Sep 28;16:1918881. doi: 10.3389/fcimb.2026.1918881 (PMC13654791; doi:10.3389/fcimb.2026.1918881)
Supplement: Supplementary file 2 [file Supplementaryfile1.docx]

Supplementary Table 1. Oligonucleotides, plasmids and bacterial strains used in the study.

Supplementary Table 2. Differential expression of genes in the KO *M.tuberculosis* strains in comparison to the wild type strain in mid-log culture.

Supplementary Table 3. Differential expression of genes in murine non-activated BMDMs infected with the KO *M. tuberculosis* strains in comparison to the BMDMs infected with the wild type strain.

Supplementary Table 4. Differential expression of genes in murine IFN-γ activated BMDMs infected with the KO *M. tuberculosis* strains in comparison to the BMDMs infected with the wild type strain.
