# Supplementary material for: Mycobacterium tuberculosis small RNAs MTS0997 and MTS1338 regulate both pathogen virulence and host immune responses
Source: Front Cell Infect Microbiol. 2026 Sep 28;16:1918881. doi: 10.3389/fcimb.2026.1918881 (PMC13654791; doi:10.3389/fcimb.2026.1918881)
Supplement: Supplementary file 3 [file Table1.docx]

**Supplementary Table 1.**

**Oligonucleotides used in the study**

| Name | Sequence, 5’-3’ |
| --- | --- |
| Oligonucleotides used for d0997 deletion mutant construction | |
| 0997_FR1_For | ATC*ggatcc*TGCAGTCGCCCGCTCGTTTTT |
| 0997_FR1_Rev | GTA*tctaga*TACCGGTGTGCCTGCCTACGG |
| 0997_FR2_For | AGT*tctaga*GCCTGCTTCGATACCAAAAATA |
| 0997_FR2_Rev | ATA*gcggccgc*CAAGGTTGCTGGGCCCGG |
| test_d0997_for | TCCGGGGTGACGTCAGGCTT |
| test_d0997_rev | AGCGAAACGTGACACCGGTT |
| Oligonucleotides used for d1338 deletion mutant construction | |
| 1338_FR1_For | TAG*agtact*AGAATGCCTTTGGTCACCAA |
| 1338_FR1_Rev | CAG*ggatcc*TTCCCCGGTATCACCAATAC |
| 1338_FR2_For | ATC*ggatcc*AGATCCTCATCCTGTTACTGCG |
| 1338_FR2_Rev | ACG*gcggccgc*TCTGTACGTCGCTGTCGGCA |
| test_d1338_for | TGGGGACGTCGGTCACGAAT |
| test_d1338_rev | CATCGCGGCGCGCTGTTCTT |
| *Restriction sites are given in italic* | |
| Oligonucleotides used for quantitative RT-PCR | |
| Rv3616c_qf | AACGATCAGTGCCATTGACGG |
| Rv3616c_qr | GCCGAACCTAACCAGCCATCA |
| Rv3615c_qf | AATCTGTGGCGATCACTCACG |
| Rv3615c_qr | CTTCGCCGCAATTCGAAGAC |
| Rv3614c_qf | ACTGCGGATCCGATTATTGGC |
| Rv3614c_qr | ACAGCTCGACATGGTCAATCC |
| Rv2485_qf | AATCTACTCGACATCTGGCGG |
| Rv2485_qr | GAGTTCCACCATCCGGCTCAT |
| Rv2450_qf | TTCCTGTCCCCGCCTGC |
| Rv2450_qr | TTGATCGACCAGTTTCCACCGG |
| Rv2429c_qf | GACACGAAATCCGCAGGTAT |
| Rv2429c_qr | CAGAGCTCGAAGTTGGCTTT |
| Rv2428c_qf | GGTTTCGATTGACAGCGAAT |
| Rv2428c_qr | GGTCGACGATAAAGGTCACG |
| Rv0867_qf | GTAAGCCCACCACATCCAAC |
| Rv0867_qr | CCCAGGTGCTTTGAGTGAAC |
| 0997_qf | GAAGCAGGCCCGGTTAGTGA |
| 0997_qr | GGCAGACCCGGCGTGACT |
| 1338_qf | GGGGAAACCCGGTGATCTG |
| 1338_qr | GGTAGGTCAAACCGGGTGTACAT |
| 16S-for | TACGTAGGGTGCGAGCGTTG |
| 16S-rev | CCCGCACGCTCACAGTTAAG |
| m_actin-for | GATCAAGATCATTGCTCCTCCTG |
| m_actin-rev | ACGCAGCTCAGTAACAGTCC |
| mIL-1β-qf | CAACCAACAAGTGATATTCTCCATG |
| mIL-1β-qr | ATCCACACTCTCCAGCTGCA |
| mIL6-qf | ACCAGAGGAAATTTTCAATAGGC |
| mIL6-qr | TGATGCACTTGCAGAAAACA |
| mIL17a-qf | GGTCAACCTCAAAGTCTTTAACTC |
| mIL17a-qr | TTAAAAATGCAAGTAAGTTTGCTG |
| mIL10-qf | TGTCAAATTCATTCATGGCCT |
| mIL10-qr | ATCGATTTCTCCCCTGTGAA |
| mTNFa-qf | CTGAACTTCGGGGTGATCG |
| mTNFa-qr | GGCTTGTCACTCGAATTTTGAGA |
| mIFNg-qf | GAGCTCATTGAATGCTTGGC |
| mIFNg-qr | GCGTCATTGAATCACACCTG |
| mIL12b-qf | CAAGAGCAGTAGCAGTTCCC |
| mIL12b-qr | GGTCCAGTGTGACCTTCTCT |
| mCXCL2_qf | GCCCAGACAGAAGTCATAGCC |
| mCXCL2_qr | GCTCCTCCTTTCCAGGTCAG |
| mIL11_qf | CCAGCTGACGGAGATCACAG |
| mIL11_qr | GGAGTAGCCGTTCCAGTCG |
| TIMP1_qf | ATGCCCACAAGTCCCAGAAC |
| TIMP1_qr | CTCAGAGTACGCCAGGGAAC |
| Loxl1_qf | CGACTATGACCTCCGAGTGC |
| Loxl1_qr | TTGAGGTTGCCGAAGTCACA |
| Adamts2_qf | CCTAGGCGTCCTGGAGGAG |
| Adamts2_qr | GCAGGATAATCCTCACCAGCA |
| Mxra8_qf | GAGGCGATGAAGGGGTGTAC |
| Mxra8_qr | CTGCTGCGCCTCCTCTAAAT |
| Col3a1_qf | CAAGGTCTTCCTGGTCAGCC |
| Col3a1_qr | GGAGCACCAGAAGGACCAG |
| Col4a1_qf | ATCCGGGACCTATGGGGC |
| Col4a1_qr | CTTGAAATCCTGGAACTCCGG |
| Col5a1_qf | GAACATTGATGCCAG CAGC |
| Col5a1_qr | TCTTGTCAGGGAAGACGCAC |
| Gzmc_qf | GGCTAAGGAGGAGACACAGC |
| Gzmc_qr | TTACCTTTCCCCAACCAGCC |
| Gzmd_qf | TCACGGCTAAGGAGGAGACA |
| Gzmd_qr | ATTGATGGACCTTGACCCCC |
| Gzme_qf | GGCCCACAACATCAAGGCTA |
| Gzme_qr | CACACATCCCCTGGCTTCAC |

**Plasmids used and generated in this study**

| Plasmid | Description | Reference or source |
| --- | --- | --- |
| p2NIL | Cloning vector; Km^r^ | [1] |
| pGOAL19 | Plasmid carrying *hyg*, *lacZ*, and *sacB* genes in a PacI cassette; Amp^r^, Hyg^r^ | [1] |
| p2NIL_d_0997 | Suicide vector to delete *MTS0997* in *M. tuberculosis*; Km^r^, Hyg^r^ | This study |
| p2NIL_d_1338 | Suicide vector to delete *MTS1338* in *M. tuberculosis*; Km^r^, Hyg^r^ | This study |
| pMV306_rrnB | pMV306hsp derivative bearing *Msm rrnB* promoter (- 79) and *rrnB* terminator,  Km^r^ | [2] |
| pMV306_0997_1338 | pMV306_rrnB derivative expressing MTS0997 and MTS1338 under *Msm rrnB* promoter*,* Kmr  *MTS0997* and *MTS1338* genes were amplified from *M. tuberculosis* genomic DNA and cloned into pMV306_rrnB at the *Bam*HI/*Hind*III restriction sites | This study |

***Mycobacterium tuberculosis* strains generated in this study**

| Strain | Description |
| --- | --- |
| d_0997 | *M. tuberculosis H37Rv* lacking *MTS0997* gene |
| d_1338 | *M. tuberculosis H37Rv* lacking *MTS1338* gene |
| dd_0997_1338 | *M. tuberculosis H37Rv* lacking both *MTS0997* and *MTS1338* genes |
| dd_0997_1338_comp | *M. tuberculosis* dd0997_1338 with integrated plasmid pMV306_MTS0997_MTS1338 (Km^r^) – sRNAs complementation |
| dd_0997_1338_empty | *M. tuberculosis* dd0997_1338 with integrated plasmid pMV306_rrnB (Km^r^) |
| wt_empty | *M. tuberculosis H37Rv* with integrated plasmid pMV306_rrnB (Km^r^) |

Amp^r^, ampicillin resistant; Km^r^, kanamycin resistant; Hyg^r^ , hygromycin.

References:

1. Parish, T.; Stoker, N.G. Use of a flexible cassette method to generate a double unmarked Mycobacterium tuberculosis tlyA plcABC mutant by gene replacement. *Microbiology* **2000**, *146*, 1969-1975.
2. Bychenko O, Skvortsova Y, Ziganshin R, Grigorov A, Aseev L, Ostrik A, Kaprelyants A, Salina EG, Azhikina T. *Mycobacterium tuberculosis* Small RNA MTS1338 Confers Pathogenic Properties to Non-Pathogenic *Mycobacterium smegmatis*. Microorganisms. **2021** Feb 17;9(2):414.
